# The Lack of a Representative Tendinopathy Model Hampers Fundamental Mesenchymal Stem Cell Research

**DOI:** 10.3389/fcell.2021.651164

**Published:** 2021-05-03

**Authors:** Marguerite Meeremans, Gerlinde R. Van de Walle, Sandra Van Vlierberghe, Catharina De Schauwer

**Affiliations:** ^1^Comparative Physiology, Faculty of Veterinary Medicine, Ghent University, Merelbeke, Belgium; ^2^Baker Institute for Animal Health, College of Veterinary Medicine, Cornell University, Ithaca, NY, United States; ^3^Polymer Chemistry and Biomaterials Group, Centre of Macromolecular Chemistry, Faculty of Sciences, Ghent University, Ghent, Belgium

**Keywords:** tendinopathy, biomaterials, tendon, *in vitro* tendon models, bioreactors

## Abstract

Overuse tendon injuries are a major cause of musculoskeletal morbidity in both human and equine athletes, due to the cumulative degenerative damage. These injuries present significant challenges as the healing process often results in the formation of inferior scar tissue. The poor success with conventional therapy supports the need to search for novel treatments to restore functionality and regenerate tissue as close to native tendon as possible. Mesenchymal stem cell (MSC)-based strategies represent promising therapeutic tools for tendon repair in both human and veterinary medicine. The translation of tissue engineering strategies from basic research findings, however, into clinical use has been hampered by the limited understanding of the multifaceted MSC mechanisms of action. *In vitro* models serve as important biological tools to study cell behavior, bypassing the confounding factors associated with *in vivo* experiments. Controllable and reproducible *in vitro* conditions should be provided to study the MSC healing mechanisms in tendon injuries. Unfortunately, no physiologically representative tendinopathy models exist to date. A major shortcoming of most currently available *in vitro* tendon models is the lack of extracellular tendon matrix and vascular supply. These models often make use of synthetic biomaterials, which do not reflect the natural tendon composition. Alternatively, decellularized tendon has been applied, but it is challenging to obtain reproducible results due to its variable composition, less efficient cell seeding approaches and lack of cell encapsulation and vascularization. The current review will overview pros and cons associated with the use of different biomaterials and technologies enabling scaffold production. In addition, the characteristics of the ideal, state-of-the-art tendinopathy model will be discussed. Briefly, a representative *in vitro* tendinopathy model should be vascularized and mimic the hierarchical structure of the tendon matrix with elongated cells being organized in a parallel fashion and subjected to uniaxial stretching. Incorporation of mechanical stimulation, preferably uniaxial stretching may be a key element in order to obtain appropriate matrix alignment and create a pathophysiological model. Together, a thorough discussion on the current status and future directions for tendon models will enhance fundamental MSC research, accelerating translation of MSC therapies for tendon injuries from bench to bedside.

## Introduction

**FIGURE 1:**
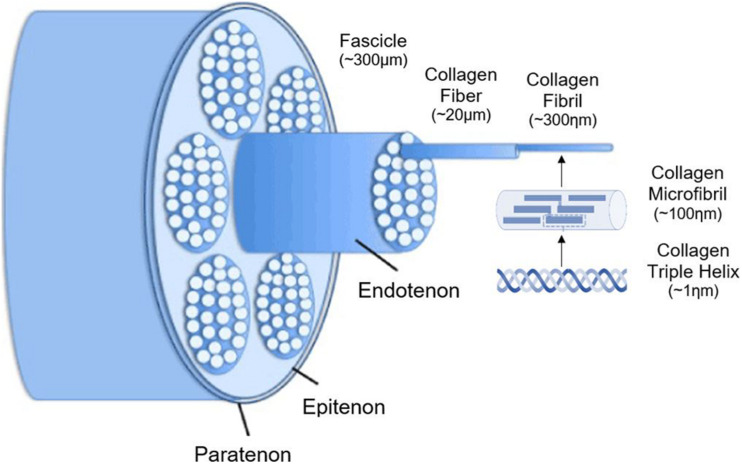
Schematic illustration of the hierarchical structure of tendons. Adjusted from Durgam and Stewart (2016) and ChemBAM (n.d.).

Tendon overuse injuries are one of the most common sports-related injuries both in humans and horses (Carpenter and Hankenson, 2004). The Achilles tendon in human patients and the superficial digital flexor tendon (SDFT) in equine patients are frequently injured structures due to their capacity to store energy during high-speed locomotion. The cumulative degenerative damage to tendons caused by high-intensity exercise and age-related microdamage might result in chronic problems of tendinopathy. Currently, injuries to the equine SDFT is the most appropriate animal model for human Achilles tendon injuries (Patterson-Kane et al., 2012; Burk et al., 2013a). Besides the chronic pain and early retirement in equine and human athletes, tendinopathy also causes economic losses and animal welfare concerns (Patterson-Kane et al., 2012). Tendons are hierarchically organized based on a triple-helix of cross-linked tropocollagen, forming insoluble collagen molecules which aggregate progressively into microfibrils, fibrils, and fibers (Figure 1). Different fibers are combined into a bundle, called “fascicles”, surrounded by endotenon. In their turn, different fascicles are grouped and surrounded by epitenon. Both endo- and epitenon supply the tendon with blood vessels, nerves and lymphatics (Wang, 2006; Docheva et al., 2015; Tan et al., 2015; Schneider et al., 2018). Tendon extracellular matrix (ECM) consists physiologically mainly of collagen I (95%), while collagen III is present in the endotenon (1–3%) (Spaas et al., 2012; Tan et al., 2015). In addition to collagen, elastin renders the tendon tissue flexible and extensible, while the ground substance in the ECM is essential for proper metabolism, shock absorption, viscoelasticity, and support (Schneider et al., 2018). Important components of the latter include proteoglycans (e.g., decorin and lumican) and glycoproteins (e.g., tenascin-C, tenomodulin, and cartilage oligomeric matrix protein). Scleraxis and Mohawk are major tenocyte-specific transcription factors which support matrix production, tenocyte proliferation, and differentiation (Liu et al., 2015). The cellular compartment of tendons consists of specialized fibroblasts, i.e., tenocytes and tenoblasts, and recently identified tendon stem/progenitor cells (Bi et al., 2007; Wang et al., 2017; Costa-Almeida et al., 2018b; Sensini and Cristofolini, 2018; Zhang et al., 2020).

Immediately following acute tendon damage, an inflammatory phase is observed, in which various inflammatory and immune cells are attracted to the injury site. Subsequently, the proliferation phase starts, characterized by fibroplasia, angiogenesis and new ECM synthesis. Finally, during the remodeling or maturation phase, tendon fibers are realigned and scar tissue is replaced by tissue-specific cells and matrix to restore native tissue properties (Spaas et al., 2012). In adult tendons, however, the healing process results in the presence of inferior scar tissue lacking the structural integrity and elasticity of the original tendon (Evans, 2012; Spaas et al., 2012; Docheva et al., 2015; Adekanmbi et al., 2017; Schneider et al., 2018; Sensini and Cristofolini, 2018; Costa-Almeida et al., 2019; Khatibzadeh et al., 2019). The limited functionality of healed tendon tissue represents a high risk of reduced performance and/or reinjury (Dyson, 2004; Spaas et al., 2012). To date, we lack knowledge on the molecular and cellular basis of tendon physiology and fail to capture essential aspects of its pathology (Nichols et al., 2019; Wunderli et al., 2020), particularly during the early stages of injury (Dakin et al., 2012). Hypocellularity and hypovascularization of the tendon may affect its ability to respond to inflammation and reduce its efficacy to repair injured tissue (Dakin et al., 2012; Nichols et al., 2019). Indeed, neovascularization occurs in response to hypoxia-associated vascular endothelial growth factor (VEGF) secretion. However, these neovessels are not completely functional and fail to deliver properly nutrients and oxygen. Consequently, the persistent hypoxia aggravates inflammation and MMP secretion, which results in further disruption of the tendon (Chung and Shum-Tim, 2012; Costa et al., 2007). Current tendinopathy treatments in humans and horses include physical therapies (cold, pressure, support, shock wave therapy, and rehabilitation programs), drug treatments (systemic or intra-lesional anti-inflammatory medication) and surgery (tenoscopy and tendon splitting), but all these therapies fail to provide tendon regeneration and restore the functionality of the original tendon tissue (Smith, 2008; Docheva et al., 2015; Ramos et al., 2019). The poor success of conventional therapy supported the need to search for novel treatments to regenerate a tissue mimicking the tendon to the greatest extent as possible (Richardson et al., 2007). Despite the great interest in MSCs due to their ability to repair tissue and reduce inflammation (Sevivas et al., 2018), common clinical applications have been hampered by several limitations. First, many experimental and pre-clinical studies have been evaluating the regenerative potential of MSCs for tendon healing and although clinical translation appears temptingly close (Pacini et al., 2007; Nixon et al., 2008; Schnabel et al., 2009; Godwin et al., 2012; Ricco et al., 2013; Smith et al., 2013; Van Loon et al., 2014), convincing evidence based on randomized, controlled, clinical studies in equine or human patients, is still lacking (Pas et al., 2017; Phelps et al., 2018; Khatibzadeh et al., 2019) and outcomes of long-term follow-up studies do not meet expectations (Geburek et al., 2017; Ahrberg et al., 2018). Second, various practical considerations regarding MSC source, dosage, administration technique, and timing, remain unanswered (Costa-Almeida et al., 2019; Shojaee and Parham, 2019). It is known that MSCs isolated from different sources display significantly diverse properties indicating potential advantages and disadvantages for the use of each MSC type in particular clinical applications (Burk et al., 2013b; Harman et al., 2020). Bone marrow, adipose tissue, and peripheral blood are the most commonly used MSC sources in equine regenerative medicine. Although adipose tissue-derived MSCs (AT-MSCs) are more easily accessible and have a higher yield after harvesting compared to bone marrow-derived MSCs (BM-MSCs), better results in treating tendon injuries are obtained with the latter (Dai et al., 2015; Zarychta-Wisniewska et al., 2019). MSCs isolated from neonatal sources, however, are reported to have a longer lifespan than MSCs isolated from adult tissues, secrete more extracellular vesicles, and show broader differentiation capacity (Burk et al., 2013b; Iacono et al., 2017; Gugjoo et al., 2019). Moreover, it has been reported that the regenerative capacity of aged cells can be restored when exposed to a young environment. These findings suggest opportunities to reverse the aging process of tissues by targeting their niche (Lui and Wong, 2020). As such, MSC-based strategies isolated from neonatal sources might represent promising therapeutic tools for tendon repair and regeneration (Sevivas et al., 2018). Third, most *in vitro* studies investigate tenogenic differentiation of MSCs while some studies explored the interaction between MSCs and tenocytes, MSCs and tendon ECM or the effects of their secretome products, but their underlying mechanisms of action have been rarely studied (Liu et al., 2017; Burk, 2019). A decade ago, Dirks and Warden reviewed the models available to study tendinopathy and concluded that a wide range of models (*in vitro, ex vivo* and *in vivo* models) is mandatory to completely understand the pathogenesis of tendinopathy. To gain insight in the underlying molecular pathways, however, *in vitro* models serve as important biological tools to study cell behavior under controlled conditions, bypassing the confounding factors associated with *in vivo* clinical trials (Dirks and Warden, 2011). Nowadays, a wide diversity of *in vitro* tendon models are used to improve our fundamental understanding of tendon mechanobiology and to study tissue replacement processes, cell-based treatments, and drug screening applications, but no generally accepted *in vitro* model exists (Butler et al., 2008; Patterson-Kane et al., 2012; Patel et al., 2017; Wang et al., 2017; Laternser et al., 2018). To the best of our knowledge, both in humans and (laboratory) animals, a generally accepted *in vitro* tendinopathy model is not available yet.

Initially, the use of MSCs for primary tissue regeneration was advocated based on their ability to migrate to and engraft in the injury site, where they would differentiate into various appropriate cell types. Nowadays, however, MSCs are considered “medicinal cell factories” secreting a variety of bioactive molecules, either in soluble form or via extracellular vesicles, with immunomodulatory, ECM modeling, trophic and anti-apoptotic activities, collectively identified as the secretome (da Silva Meirelles et al., 2009; Manning et al., 2015; Presen et al., 2019; Zhang et al., 2020). Indeed, MSC-conditioned medium (MSC-CM), which includes all their secretome products, has similar regenerative effects as MSCs, which illustrates the impact of these secretome products (Phelps et al., 2018). It has also been demonstrated *in vitro* that cell proliferation and migration of injured tenocytes were promoted after MSC-CM administration (Chen et al., 2018; Li et al., 2020). Nevertheless, the regenerative capacities of MSCs are multifaceted and strongly cell- and tissue context-dependent, and the insights into their trophic and protective mechanisms in the context of tendon therapies remain scarce (Pas et al., 2017; Vizoso et al., 2017; Bogatcheva and Coleman, 2019; Burk, 2019; Harrell et al., 2019; Presen et al., 2019; Al Naem et al., 2020). As there is an urgent need to (i) unravel the MSCs’ mechanisms of action, (ii) find a therapy that consistently yields positive results, (iii) investigate the most optimal treatment protocol, (iv) unravel the wide potential of the MSCs’ bioactive factors, and (v) identify the most appropriate MSC source, an optimal *in vitro* tendon model might provide a lot of answers. Moreover, by establishing a state-of-the-art physiologically representative *in vitro* tendinopathy model, the use of experimental animals will be drastically reduced. The effect of potential therapies for tendinopathy, with great emphasis on MSCs and their secretome, can be studied *in vitro*, and answers to questions relevant for clinical applications (like timing of treatment, dosage, immunomodulatory activities, etc.) can be provided, further reducing the number of *in vivo* experiments considerably.

**FIGURE 2:**
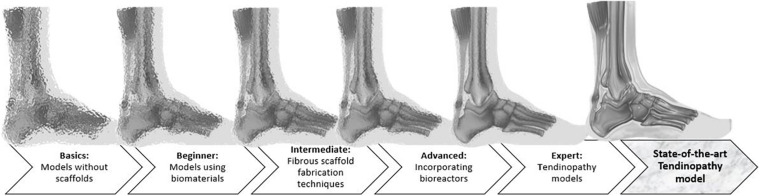
Illustration of the review’ structure, starting from very basic but very unclear, blurred models to increasingly complex models, which are more appropriate to represent an injured Achilles tendon and to clarify the MSC mechanisms of action.

An overview of the models available to mimic tendon tissue *in vitro* with increasing complexity is given throughout the review (Figure 2). Elements to establish a representative *in vitro* tendinopathy model are suggested, including techniques which might be promising but are not yet optimized to incorporate in a tendon model. Rather than citing all available literature on tendon tissue engineering, models are discussed with an emphasis on their strengths and shortcomings regarding fundamental research on the regenerative capacities of MSCs. Therefore, the evaluation criteria incorporated in this review are i) representative cellular phenotype (of tenocytes, tenogenic-differentiated MSCs, fibroblasts, or tendon stem/progenitor cells) as demonstrated by the spindle-shape morphology and tenocyte marker expression, (ii) production of ECM (evaluated by gene expression and immunohistochemistry) and allowing cell–matrix interactions, (iii) supporting nanometric and axially aligned structure (anisotropy), (iv) responsive to physiological levels of uniaxial strain, (v) neuro-vascular supply, and (vi) mimicking micro-damage, like acute injuries and chronic overuse.

## Basics: Models Without Scaffolds

### Two-Dimensional (2D) Models

**FIGURE 3:**
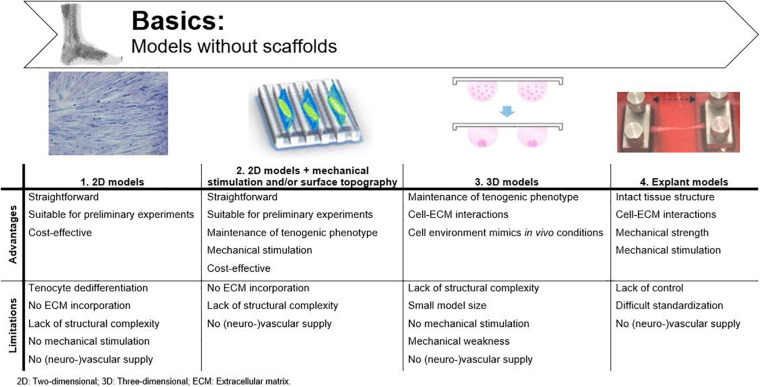
Advantages and disadvantages of basic tendinopathy models without scaffolds. Adjusted from Krishna et al. (2016); Ryu et al. (2019) and Tohidnezhad et al. (2020).

2D cell cultures are commonly used to study cell behavior. In such a simplified environment, basic morphology, gene expression, and differentiation are easily studied without confounding factors (Laternser et al., 2018). However, mimicking the biomechanical and -chemical environment of native tendon is crucial when studying the behavior and mechanisms of action of MSCs (Grier et al., 2017; Al Naem et al., 2020). A common issue in 2D tenocyte cultures is dedifferentiation. With increasing cell passage, tenocytes lose their characteristic spindle-like morphology and consequently, their functionality (Yao et al., 2006). Their changing morphology is accompanied by a significant decrease in collagen I and tenomodulin mRNA expression, as demonstrated in the study of Zhu et al. (Zhu et al., 2010). Unlike *in vitro* cultured tenocytes, tenocytes *in vivo* are not organized in confluent sheets and are able to actively interact with the ECM (Patterson-Kane et al., 2012; Laternser et al., 2018). Therefore, 2D cultures are no longer used for tissue functionality or regeneration studies. However, these models are still useful for setting up preliminary experiments, implementation as control condition or, for example, investigating cytotoxic effects (Occhetta et al., 2013; Fessel et al., 2014). Because of their simplicity, 2D models are more cost-effective than the sophisticated techniques explained below (Figure 3) (Wunderli et al., 2020).

### 2D Models Combined With Mechanical Stimulation and/or Surface Topography

Contact-guidance might offer a solution to maintain tenocyte differentiation in 2D cell cultures. Mechanical stimulation and surface topography of cell culture surfaces influence cell density, cellular alignment, and the organization of newly deposited matrix (Nikolovski et al., 2003; Park et al., 2006; Zhang et al., 2021; Kuo et al., 2010). *In vivo*, it is known that mechanical stimulation is important to maintain tendon homeostasis (Screen et al., 2005). However, cultured cells *in vitro* also respond to mechanical strain by displaying a more spindle-shaped morphology and adjusting their DNA synthesis toward the production of collagen I (Figure 3) (Wang et al., 2003; Matheson et al., 2006). As such, Riboh et al. (2008) induced the tenogenic phenotype in epitenon tenocytes, sheath fibroblasts, bone BM-MSCs and AT-MSCs by exposing the cells to intermittent cyclic strains (4%, 0.1 Hz, 1 h on/2 h off). To study the impact of surface topography on cellular alignment and tenocyte characteristics, Kapoor et al. (2010) used grooved substrates with different diameters (50–250 μm) to verify the influence of physical parameters on tenocytes. Both cell density and cellular alignment were affected by the microtopography of the substrates, with 50 μm grooves having the most pronounced impact, as demonstrated by denser and more longitudinally oriented collagen fibers. No significant impact was observed on matrix gene expression or cell phenotype (Kapoor et al., 2010). However, when micro-grooved silicone surfaces were combined with cyclic uniaxial stretching, human tenocytes showed a phenotype comparable to the *in vivo* situation and an increased cellular production of collagen type I in a stretching-magnitude-dependent manner (4 and 8% stretch) (Wang et al., 2003; Yang et al., 2004). Despite the improved knowledge on cell proliferation and cellular alignment, these modified 2D cell cultures still lack structural complexity to mimic tendinopathy *in vitro*.

### Three-Dimensional (3D) Models

Apart from contact-guidance, tenocyte dedifferentiation can also be countered by spheroid formation. As such, a 3D set-up was developed using hanging drop cultures, in which tenocytes are exposed to microgravity (Theiss et al., 2015). Theiss et al. (2015) and Kraus et al. (2017) generated equine tenocyte spheroids when specific growth factors were supplemented to the culture medium. They observed that tenocytes within spheroids better preserved their spindle-like morphology and showed enhanced expression of tenogenic genes like collagen I, collagen III, and scleraxis and expression of the chondrogenic transcription factor *SOX9* (Theiss et al., 2015; Kraus et al., 2017). Spheroids provide the functionality which is lacking in 2D cultures, and as such, are more suitable to study pathological conditions *in vitro* (Figure 3) (Laternser et al., 2018). Calve et al. (2004) were the first to engineer viable tendon tissue constructs *in vitro* without using artificial scaffolds. They created these constructs by allowing self-assembly of isolated rat Achilles tendon tenocytes into a cylinder, which resembled embryonic tendon consisting of collagen fibrils, many tenocytes and a non-collagenous ECM. Moistening of the constructs was provided by bathing each sample individually in culture medium. Both for spheroids and the self-assembled tenocyte cylinders, cell–cell and cell–matrix interactions can be studied as the cultured tenocytes produce ECM. Therefore, to extrapolate *in vitro* results to *in vivo* clinical trials, it would be of great benefit to study MSCs in 3D environments (Burk, 2019), as for example the immunomodulatory potential of the MSCs is shown to be altered in 3D vs. 2D (Follin et al., 2016; Li et al., 2018). The disadvantages of these techniques are the small model size and the low mechanical properties of the constructs when compared to mature tendons due to the rather immature morphology of the tenocytes and the lack of mechanical stimulation during the culture period (Calve et al., 2004).

### Explant Models

Another approach to study tendon tissue is by using tendon explant models. Intact native tissue samples can be dissected and cultured *ex vivo*. The main advantage of this method is the intact tissue architecture, which allows studying cell-ECM interactions in a near-physiological environment. These models have been used for structure and function characterization of tendon tissue, to study cell-mediated processes and to investigate crosstalk mechanisms (Wunderli et al., 2020). “Clamp and stretch” models of these explants are often implemented to define mechanical characteristics of tendon tissue (Goldstein et al., 1987). This technique is characterized by the application of mechanical load in a longitudinal manner on a tissue sample clamped at both ends, while the deformation and applied forces are monitored (Dyment et al., 2020). Many different bioreactors have been developed for mechanical stimulation of cell and tissue cultures, which are described in more detail in the section “Advanced: Incorporating bioreactors”. The most frequently described problems of longitudinal stretch systems are the rather heterogeneously transmitted strain, along with grip slippage due to the high mechanical forces used (Figure 3) (Brown, 2000). Basic “clamp and stretch” studies often cover a small-time interval because of the lack of nutrient supplementation, resulting in dry, non-physiological circumstances. Devkota and Weinhold (2005) developed an advanced tissue explant system to create and monitor mechanical changes occurring with tendon overuse. The machine can be placed in a standard incubator and is equipped with video strain analysis capabilities for monitoring. Another improvement over previous models is the use of load-controlled operation, preventing grip slippage (Devkota and Weinhold, 2005). The incorporation of mechanical stimulation mimics the *in vivo* load-bearing function. Explant models as such can provide useful insight into tendon (patho-)physiology and have also previously been used to study MSC characteristics. Costa-Almeida et al. (2018a) studied the communication between AT-MSCs and native tendon ECM in a *trans*-well tendon explant model. Although the AT-MSCs were not directly in contact with the tendon explant, significant changes in MMP secretion, collagen III and tenascin-C deposition were monitored when AT-MSC were co-cultured compared to single tenocyte cultures, suggesting ECM remodeling. The authors proposed explant co-cultures as a tool to unravel cellular communication and tendon healing (Costa-Almeida et al., 2018a). Wunderli et al. (2020) excessively reviewed tendon explant models for physiologically relevant *in vitro* studies and confirmed their suitability for investigating cellular cross-talk. Furthermore, Youngstrom et al. (2015) reseeded decellularized tendon scaffolds with BM-MSCs to evaluate the effects of different strain protocols on ECM composition, gene expression and mechanical properties of the scaffolds. The goal of these experiments, however, was to validate the custom-designed bioreactor and not specifically to characterize MSCs (Youngstrom et al., 2015). Nevertheless, explant models are subjected to variable conditions which are not controllable enough to obtain reproducible results when aiming to elucidate exact MSCs’ mechanisms of action (Wunderli et al., 2020).

## Beginner: Models Using Biomaterials

**FIGURE 4:**
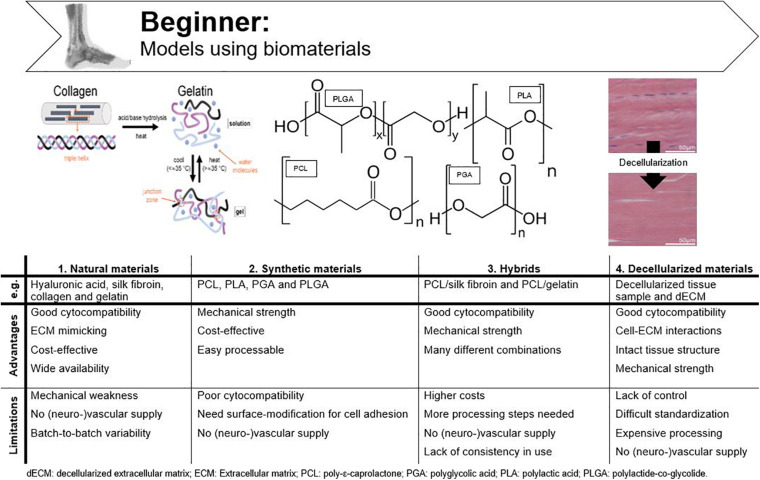
Advantages and disadvantages of tendinopathy models incorporating biomaterials. Adjusted from Barrett et al. (2013) and ChemBAM (n.d.).

Biomaterials are defined as any material that is able to interact with biological systems and can consist of natural and/or synthetic materials (National Institute of Biomedical Imaging and Bioengineering, n.d.). While standard cell culture materials do not resemble physiological circumstances, e.g., native ECM, biomaterials are specifically designed to deliver mechanical, structural, and compositional stimuli to the cells (Caliari and Burdick, 2016). Most requirements for biomaterials are based on demands for tissue engineering. In relation to an *in vitro* tendon model these include (i) correct biochemical composition and structure, (ii) biocompatibility toward appropriate cell population, (iii) appropriate mechanical strength and elasticity to mimic cell-microenvironment interactions of *in vivo* tendon tissue, and (iv) an easily processable material (Ma, 2004; Kuo et al., 2010; Rodrigues et al., 2013). An overview of the advantages and disadvantages of the discussed materials is given in Figure 4.

### Natural Materials

Currently used natural materials for tendon applications are hyaluronic acid, silk fibroin, collagen and gelatin. The general advantage of using natural materials for tissue engineering is the good cytocompatibility due to the functional chemical groups available for cellular binding. For example, the tripeptide arginine-glycine-aspartic acid (RGD) sequence functions as integrin-binding sites, which are of critical importance for cell adhesion (Ruoslahti, 1996). For tissue engineering, it is important that cells delivered through the scaffold remain in place, but also for an *in vitro* model, it is of great importance that cultured cells can interact with the biomaterial.

Collagen I is the most extensively used natural material because of its low cost and high physiological prevalence in tendon tissue (Kuo et al., 2010; Caliari and Burdick, 2016; Wu et al., 2018). Although collagen gels, sponges and extruded fibers are being used for tendon tissue engineering, their main drawback is mechanical weakness (Qiu et al., 2016; Lake et al., 2020). Cheng et al. (2008) were able to upregulate the mechanical strength of collagen (30-fold) by producing electrochemically aligned collagen (ELAC) bundles. When these bundles were seeded with MSCs, the expression of tendon-specific genes (scleraxis and tenomodulin) was upregulated when compared to randomly oriented collagen threads, which illustrates that collagen might be used to replace tendon (Kishore et al., 2012; Shimada et al., 2014). As with all natural materials, another disadvantage of extracted collagen is batch-to-batch variability, which can be circumvented by using recombinant collagen (Slaughter et al., 2009; Tytgat et al., 2019). Gelatin is denatured collagen which can be used as an ECM mimic after chemical modification and crosslinking to provide stability at elevated temperatures and which is less immunogenic when compared to collagen (Laternser et al., 2018; Van Hoorick et al., 2019). Other advantages are low cost and wide availability, especially when considering large-scale *in vitro* studies (Van Hoorick et al., 2019). Furthermore, we recently demonstrated the excellent biocompatibility of cross-linked gelatin (gelatin-methacrylamide and gelatin-norbornene) to support equine tenocyte cultures (Meeremans et al., 2021). Silk fibroin is a worthy alternative to collagen and gelatin for tendon and ligament tissue engineering and is collected from silkworms, mostly *Bombyx mori*. The advantages of silk are its strong mechanical characteristics, good cytocompatibility, easy processability, and its potential to be changed into many different forms (Minoura et al., 1995; Rockwood et al., 2011; Yao et al., 2016). In the study of Chen et al. (2010), knitted silk-collagen scaffolds were used in which seeded MSCs showed good adherence to the scaffold, proliferated well, and showed tendon biocompatibility after mechanical stimulation. Tenogenic differentiation of MSCs, characterized by adopting a tenocyte-like shape and expression of tendon-related genes, illustrates the suitability of silk for supporting tenocyte cultures and tendon tissue engineering. Hyaluronic acid belongs to the group of glycosaminoglycans and is often implemented in tendon tissue engineering to increase the mechanical strength (Liu Y. et al., 2008). Funakoshi et al. (2005) fabricated a 3D chitosan/hyaluronic acid scaffold to repair tendon defects in an *in vivo* rabbit model. This newly designed scaffold had previously shown potential as a biomaterial for cartilaginous tissue scaffolds and was, therefore, hypothesized to enhance collagen I production when implanted in *in vivo* tendon defects. This study found that in addition to the enhanced collagen production, the mechanical strength of the regenerated tendons also increased when seeded with fibroblasts, displaying potential for an *in vitro* tendon culture (Funakoshi et al., 2005; Yamane et al., 2005).

### Synthetic Materials

Synthetic materials are also widely used in tissue engineering as they provide excellent mechanical support, are easily processable, and are cost-effective. Synthetic polymers applied in tendon tissue engineering are e.g., polylactic acid (PLA), polyglycolic acid (PGA), and their copolymers such as polylactide-co-glycolide (PLGA) (Ouyang et al., 2003; Cao et al., 2006; Chen et al., 2010; Yang et al., 2016; Aldana and Abraham, 2017; Wu et al., 2018). All belong to the group of polyesters and are attractive for *in vivo* use due to the formation of natural metabolites upon degradation. However, because of the hydrophobic nature of polyesters, cell adhesion is far from optimal and their *in vitro* application is less attractive (Liu Y. et al., 2008). Cao et al. (2006) were able to generate tendon tissue *in vitro* by culturing tenocytes on PGA fibers arranged into a cord-like construct, both with and without application of constant strain (two groups). They found that the generated tissue resembled natural tendon tissue histologically in both groups, as opposed to the cell-free control group. The application of constant strain improved mechanical characteristics but was detrimental for scaffold thickness and collagen fiber alignment, and thus, considered suboptimal. The authors suggested that aligned fibers instead of non-woven fibers should be used preferably and that the strain regime for mechanical load should be intermittent instead of constant (Cao et al., 2006). The fact that polymer fibers should be aligned to mimic the highly organized collagen fibers, was further corroborated by a study of Lee et al. (2005), where they developed tissue-engineered ligaments of polyurethane. Tendons and ligaments indeed have some features in common such as the hierarchical structure and the non-linear mechanical properties (Sensini and Cristofolini, 2018). Moreover, when aligned PLA scaffolds were used, Yin et al. (2010) showed that tendon stem cells displayed a spindle-shape morphology and tendon-like tissue was formed. Ouyang et al. (2003) and Sahoo et al. (2006) compared different PLGA production technologies to create a tendon/ligament biodegradable scaffold. Both *in vivo* and *in vitro* studies described favorable BM-MSC morphology (spindle-shape) and alignment (Ouyang et al., 2003; Sahoo et al., 2006). Synthetic polymers are often implemented for their superior mechanical strength but they lack functional chemical groups for cellular binding and often need surface modification (Kuo et al., 2010). Locke et al. (2020) recently assessed the use of synthetic polyethylene glycol (PEG) hydrogels. To create a material suitable for tendon regeneration, a degradable linker peptide, a multifunctional collagen mimetic peptide and integrin-binding peptide sequences were incorporated into the hydrogel (Locke et al., 2020). Although the authors highlighted the potential of such multifunctional and synthetic hydrogels for tissue regeneration, especially when considering their mechanical properties, the approach is rather complex to (repeatedly) implement in an *in vitro* model.

### Hybrids

An *in vitro* tendon model needs comparable mechanical strength as natural tendon to mimic the physiological stiffness the cells experience *in vivo*, but also specific surface stimuli for cell proliferation, hybrids are preferred for tendon tissue engineering. Hybrid scaffolds consist of various synergistically combined natural and synthetic polymers (Kuo et al., 2010). As previously mentioned, a knitted collagen-silk scaffold was suitable for tenogenic differentiation of human MSCs, but no comparison with other scaffolds was made in that particular study (Chen et al., 2010). Liu H. et al. (2008) seeded a combined scaffold of knitted silk and microporous silk sponge with BM-MSCs for (anterior cruciate) ligament tissue engineering and successfully overcame limitations of individual designs. Poly-ε-caprolactone (PCL), a synthetic biomaterial is often used in tissue engineering because of its biocompatibility, low cost and slow degradation. However, its hydrophobic nature prevents efficient cell attachment and the use as mono-material for tendon tissue engineering or for *in vitro* model design is, therefore, not recommended (Yang et al., 2016; Chen et al., 2017). Chen et al. (2017) combined PCL and silk fibroin in aligned scaffolds for supporting dermal fibroblast attachment and guidance of cell proliferation along the orientation of the nanofibers. By combining both materials, a new superior nanofiber scaffold for tendon tissue engineering was produced (Chen et al., 2017). Since the dermal fibroblasts differentiated into tenocytes (Chen et al., 2017), a similar response is in our opinion to be expected after tenocyte seeding and thus this strategy is suitable for *in vitro* tendon design. A multi-layered PCL/gelatin scaffold for tendon tissue engineering was designed by the group of Yang. The scaffolds seeded with AT-MSCs were found to mimic the native tendon tissue structure, mechanical properties and cell phenotype (Yang et al., 2016).

### Decellularized Constructs

Instead of using polymers and complicated chemical production technologies, decellularized tendons are also widely used for tissue engineering (Lake et al., 2020). Decellularization protocols often use detergents to solubilize cell debris of tendon tissue samples aiming to remove all immunological signals. These constructs provide the same mechanical properties and integrin binding sites as natural tendon tissue, allowing implementation as a biological graft material *in vivo* and creating new opportunities for fundamental research models (Kuo et al., 2010). However, the amount of removed cells, DNA, immunological signs, and mechanical characteristics are depending on the used decellularization process (Kuo et al., 2010; Youngstrom and Barrett, 2016). When decellularization is combined with chemical oxidation, mechanical characteristics of the tendon extracts are preserved and all DNA is withdrawn (Whitlock et al., 2007). In a study of Youngstrom and Barrett (2016) decellularized equine SDFT was stated as ideal for tendon tissue engineering because of the preservation of the biochemical composition, structure and mechanics of native tendon (Barrett et al., 2013). Tissue sample decellularization can also be combined with enzymatic digestion to create soluble decellularized ECM (dECM), which can be analogously processed like natural and synthetic materials, or even combined with them (Santschi et al., 2019). When developing an *in vitro* model, however, the exact dECM composition should be determined every time in order to obtain reproducible results, which results in an undesirably expensive and cumbersome production process.

## Intermediate: Fibrous Scaffold Fabrication Techniques

It is important to emphasize different production processes, as scaffold properties are strongly influenced by the used processing techniques and applied parameters (Grier et al., 2017). For example, collagen gels are mechanically very weak, but their strength can be influenced by different crosslinking methods. Maximal collagen strength could be reached by physical crosslinking using dehydrothermal and ultraviolet light, but this came at the cost of decreased migration of dermal fibroblasts (Cornwell et al., 2007). Hydrogels, a water-swollen network of polymers, have emerged as the most promising out of the different biomaterial systems (Caliari and Burdick, 2016). The main advantage of hydrogels is their large water content, mimicking the hydrophilic nature of (tendon) tissue ECM (Yang et al., 2016; Schneider et al., 2018). A disadvantage is the typically weak mechanics, which need improvement by various chemical modifications required for crosslinking.

**FIGURE 5:**
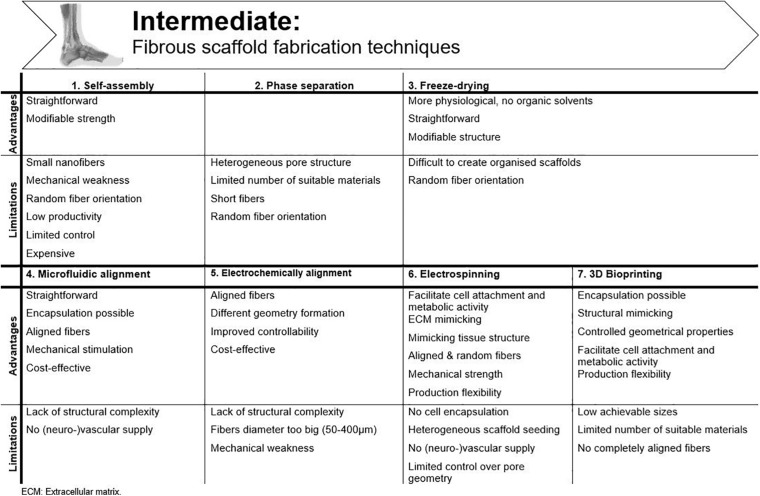
Advantages and disadvantages of the available fibrous scaffold fabrication techniques for tendon tissue engineering.

Important requirements for 3D constructs are spatiotemporal control of the 3D cellular microarchitecture and ECM distribution (Lu et al., 2013). Available literature underlines the advantage of aligned nanofibers over randomly oriented fibers to mimic tendon tissue. However, ideal fiber characteristics, such as diameter, pore size, spacing and angle are still under debate. The oldest production techniques use a “top-down” approach, in which cells are seeded onto a designed scaffold. After proliferation, the seeded cells need to produce new ECM. Complex functional tissues are hard to design top-down and size is confined by diffusion limitations (e.g., oxygen diffusion, 100–200 μm) (Radisic et al., 2006; Loh and Choong, 2013; Lu et al., 2013; Richards et al., 2016). By “bottom-up” engineering, microscale tissue building blocks with specific micro-architecture are carefully assembled together to build larger constructs. Different building blocks can consist of different cell populations and biomaterials, creating micro-organs (Lu et al., 2013). Another classification can be made regarding conventional methods vs. additive manufacturing technologies. A more detailed overview of the techniques discussed is shown in Figure 5.

### Self-Assembly

In this bottom-up method, very small nanofibers (<100 nm to a few nm) are produced through weak interactions. The productivity of this technique is rather low and the process is only under limited control (Alghoraibi and Alomari, 2018). Cornwell et al. (2007) improved the mechanical strength of self-assembled collagen threads, formed by extrusion into a bath of fiber formation buffer, by dehydrothermal treatment and ultraviolet crosslinking. Another way to improve strength is by administering strain prior to fiber drying. Through this way, the ultimate tensile strength (UTS) in pre-stretched fibers is reported to be five times higher (Kew et al., 2011). The research group of Attenburrow compared different co-agents to produce extruded collagen fibers. They aimed to preserve the advantages of extruded collagen fibers and, at the same time, upregulate production and stability. Both polyethylene glycol and NaCl are considered highly suitable to reconstruct collagen fibers (Zeugolis et al., 2008a, b). Due to the poor mechanics of collagen, the formation of stable 3D constructs is not yet achieved, and the high costs associated with this technique hamper research and practical applications (Lu et al., 2013). Another disadvantage of collagen extrusion techniques is their limited efficiency as the production process is very time consuming (Kew et al., 2011).

### Phase Separation

Phase separation is generated by introducing a non-solvent. Physical incompatibility between polymer and solvent results in separation and formation of nanofibers after gelation. However, these scaffolds have a heterogeneous pore structure, which makes them unsuitable for tissue engineering (Lu et al., 2013). Moreover, the porosity needs to be interconnected to allow cell infiltration and diffusion of culture media (Freyman et al., 2001). An alternative is thermally induced phase separation, but only few (synthetic) polymers are suitable for this process and short fibers are obtained, limiting the *in vitro* model capability (Alghoraibi and Alomari, 2018).

### Freeze-Drying

Using this technique, the polymer is frozen (−80°C) to obtain a porous structure, after which the formed ice crystals are sublimated (De France et al., 2018). This process is more appropriate for biomedical applications due to the lack of organic solvents and the more simple method, yet it is difficult to obtain hierarchically organized scaffolds with this method (Alghoraibi and Alomari, 2018). Collagen freeze-drying is regularly used to create skin regeneration scaffolds, but too little structure is present to consider this approach for tendon applications (Freyman et al., 2001). Caliari and Harley (2011) provided alignment by adding a collagen-glycosaminoglycan melt to a polytetrafluoroethylene-copper mold before freeze-drying. Equine tendon cells showed increased attachment to the surface, metabolic activity, and cell alignment, when compared to an isotropic (non-aligned) scaffold. By including directional solidification, contact guidance cues were implemented and a more physiologically relevant model was created (Caliari and Harley, 2011). This model was also utilized by Grier et al. (2017) to study the influence of pore size and stiffness on tenocyte metabolic activity and gene expression. Increased tenogenic gene expression and cell activity was seen for higher crosslinking densities and smaller pore sizes (Grier et al., 2017).

### Microfluidic Alignment

While contact guidance in 2D cultures is provided by culture plastic surface modifications (see section “Two-Dimensional (2D) Models” above), microfluidic alignment is a popular method in 3D cultures to create scaffolds with well-defined geometry. By collagen polymerization inside channels (often in polydimethylsiloxane, PDMS) fiber alignment is realized. Lee et al. (2006) examined the effect of different channel widths on alignment. They considered this technique suitable for cell studies on movement, signaling, growth and differentiation pathways, although it must be mentioned that they did observe a reduced alignment efficiency of threads with increasing channel width, resulting in a greater angle between fibers (Lee et al., 2006; Uquillas et al., 2012). Occhetta et al. (2013) combined micro-molding within PDMS stamps and hydrogel photopolymerization to establish an *in vitro* model. Instead of seeding, they encapsulated BM-MSCs and endothelial cells in the hydrogel, which is much more efficient, more physiologically relevant and more compatible with vascular supply (Occhetta et al., 2013; Waheed et al., 2019).

### Electrochemical Alignment

An electrical current can be applied to the collagen solution to produce densely packed, aligned fibers, the so-called ELAC threads, as already mentioned earlier. Advantages of this technique are low cost, different geometry formation by changing electrode geometry and improved controllability (Uquillas et al., 2012). Cheng et al. (2008) were the first to produce ELAC threads. Collagen bundles were formed with an average diameter of 50–400 μm, which is much larger than the fiber diameter achieved with self-assembly. Tenocytes seeded on ELAC threads were able to survive and proliferate on the bundles. However, only half the strength of native tendon was reached (Cheng et al., 2008). The group of Uquillas was able to increase the mechanical strength by crosslinking ELAC with 2% genipin (Uquillas et al., 2012). However, to increase biomimetic fibril structure, a smaller diameter is desirable (±100 nm range) (Smith, 2008; [Bibr B98][Bibr B225]).

### Electrospinning

#### Non-modified Electrospinning Structures

Using electrospinning, ultrafine fibers within nanometer range are produced which enables tendon tissue engineering applications. The micro- and nanostructure can be precisely designed by altering process parameters and polymer solution (De France et al., 2018). Fibers are produced through subjecting natural or synthetic materials in a syringe pump to a high-power electrical field, surface tension is overcome by electrostatic forces, and a jet of fluid is ejected onto a grounded collector (Zhong, 2016). Electrospun nanofibers have a high surface area to volume ratio, mimic the tendon ECM and, therefore, facilitate cell attachment and metabolic activity (Sahoo et al., 2006). Many derivative methods of traditional electrospinning have been developed in which highly anisotropic structures are created, thus, applicable for tendon applications, such as wet spinning, melt electrospinning, or coaxial electrospinning. In wet electrospinning, the usual metal collector is replaced by a liquid bath. If the polymer substance is heated during extraction, it is called melt electrospinning. A cylindrical collector is typically used, resulting in more aligned fibers when compared to a traditional setup (Alghoraibi and Alomari, 2018; Sensini and Cristofolini, 2018). Coaxial electrospinning combines two polymer solutions in a core-shell setup. For a more comprehensive technical description of the various techniques, we refer to other reviews (Alghoraibi and Alomari, 2018; Sensini and Cristofolini, 2018).

In various electrospinning studies, scaffolds are evaluated for tendon tissue engineering by the (un)successful tenogenic MSCs differentiation. For example, James et al. (2011) used PLGA to compare an electrospun matrix with a 2D film. Enhanced collagen I expression was observed after growth factor supplementation in both matrices, but scleraxis expression was only upregulated when AT-MSCs were cultured on the electrospun matrix. The tensile strength of the scaffold was within the range measured for regular human flexor tendons though not sufficient for those perceived by Achilles tendons (Sensini and Cristofolini, 2018). Full-thickness cell infiltration was evaluated in multi-layered aligned electrospun PCL matrices by Orr et al. (2015). In that study, it was demonstrated that fiber alignment has no positive effect on ECM production and cell infiltration after 28 days of culture, but tenomodulin expression by AT-MSCs and mechanical properties were increased (Orr et al., 2015). Yang et al. (2016) combined PCL and methacrylated gelatin onto a rotating vessel. Using these scaffolds, ECM structure and mechanical anisotropy were mimicked, and tenocytic differentiation of AT-MSC was induced. In the study of Ramos et al. (2019), in which PCL was combined with cellulose acetate, decreased fiber diameter and tensile strength were observed with increasing cellulose acetate concentrations. The latter illustrates that the balance between the hydrophobic nature of PCL and the hydrophilic nature of cellulose acetate is difficult to realize in order to promote cell interactions and, at the same time, provide sufficient strength.

Natural materials are less frequently used as a mono-material in electrospinning. Ghiasi et al. (2014) designed nano-coated textured silk yarns for tendon and ligament scaffold application. A better surface roughness was achieved, resulting in a more porous surface to support cell migration to the inner part of the scaffolds. Unfortunately, the mechanical strength of the constructs was insufficient (Ghiasi et al., 2014). Tu et al. (2013) combined a PLA core with a collagen sheath to create aligned fibers with high mechanical strength and adequate biocompatibility. PLA/PCL/collagen scaffolds were fabricated by Xu et al. (2014), by using a dynamic water flow system for electrospinning. When tendon stem cells were seeded onto the scaffolds, they showed good proliferation and increased gene expression in response to mechanical stimulation (Xu et al., 2014).

#### Textile Manufacturing Techniques

To reconstitute filamentous collagen structures and improve the mechanical strength, electrospinning configurations have been modified to produce bundles and yarns and have been combined with different textile production methods such as braiding, knitting, and weaving. Sahoo et al. (2006) designed a nano-microfibrous polymer tendon scaffold by electrospinning PLGA onto a knitted PLGA scaffold, as such bypassing the poor cell seeding results associated with braided fabrics. Although favorable cell properties were obtained, mechanical properties were far from comparable with native tendon (Sahoo et al., 2006). Barber et al. (2013) braided three, four, or five, aligned bundles of electrospun PLA nanofibers. The three-bundle braided scaffold showed superior mechanical characteristics compared to the other groups. Tenogenic differentiation of MSCs on the three-bundle scaffolds was observed after supplementation of tenogenic growth factors and upon applying cyclic tensile strain, highlighting the *in vitro* potential (Barber et al., 2013). Braiding, however, results in tightly packed biomaterials, having a negative impact on cell proliferation and infiltration (Laranjeira et al., 2017). Czaplewski et al. (2014) investigated the effect of fiber chemistry and braiding angle on the scaffold’s mechanical properties and tenogenic differentiation using human induced pluripotent stem cells. They showed that large angles, thus less dense packaging, better supported tenogenic differentiation (Czaplewski et al., 2014). Vuornos et al. (2016) compared different medium compositions, biomaterials and scaffold structures in order to identify the most efficient set-up to produce tendon-like matrix *in vitro*. When compared to foamed PLA/PCL scaffolds, braided PLA scaffolds were superior both for cell characteristics and tenogenic differentiation of AT-MSCs. Moreover, the PLA scaffolds expressed a similar elastic modulus as native Achilles tendons (Vuornos et al., 2016).

#### Hybrids

Both production technologies and biomaterials mentioned above can be extensively combined to establish the ideal *in vitro* tendon model. However, it is mandatory that the structure does not become too complex to be generally accepted and stays controllable. Each single component should be evaluated for necessity and added value. Wang et al. (2018) designed a novel scaffold for tendon regeneration by combining a PCL shell with an electrospun PCL/polyethylene oxide core. The scaffold had tendon-like mechanical properties and the cultured tenocytes expressed a higher amount of phenotypic markers when compared to isotropic control scaffolds (Wang et al., 2018). Despite the achieved mechanical strength, they were not able to mimic native ECM. As the synthetic polymers used in this model did not contain the functional chemical groups available for cellular binding, this setting is deemed not suitable as a good *in vitro* tendon model. The group of Rinoldi et al. (2019) combined coaxial extrusion printing and wet spinning of a bioink composed of alginate and methacrylated gelatin, which allows cell encapsulation. Tenogenic differentiation of BM-MSCs and aligned cell/fiber orientation was successfully achieved (Rinoldi et al., 2019). Laranjeira et al. (2017) designed an artificial tendon construct consisting of continuously aligned nanofiber threads (PCL/chitosan) reinforced with cellulose nanocrystals. Biocompatibility, cell elongation, and anisotropic organization was assessed after seeding tenocytes and AT-MSCs. Tenogenic differentiation of AT-MSCs was reached and tenocyte dedifferentiation prevented (Laranjeira et al., 2017). Unfortunately, vascularization was not provided which hampers extensive use of this model. Wu et al. (2017) produced a nanofibrous, woven biotextile, made of electrospun PCL yarns interlaced with PLA multifilaments. The woven scaffolds had a significantly larger pore size and showed better mechanics than the non-woven controls. Tenocyte and AT-MSCs proliferation and gene expression were upregulated in the woven scaffolds. Subsequently, after supplemental seeding of human umbilical vein endothelial cells (HUVECs) on the woven scaffolds with AT-MSCs and tenocytes, tenogenic gene expression further increased (Wu et al., 2017). Although vascularization building-blocks are included in this model, the exact function of the HUVECs was not evaluated in this study.

More than one hundred scientific papers and several reviews have described different electrospun nanofibers which can be used for tendon tissue engineering because of their ability to mimic ECM structure and its production flexibility (Sensini and Cristofolini, 2018). However, current models still require some optimization before a standalone model can be established. Most electrospinning set-ups are not suitable for cell encapsulation, which is more physiologically relevant and significantly more compatible with the incorporation of vascular supply than cell seeding (Waheed et al., 2019). In addition, effective vascularization has not yet been achieved in electrospun scaffolds and homogeneous seeding is often difficult (Merceron et al., 2015). The search for the ideal material (or combination of materials) is still ongoing. A combination of PCL and chemically modified collagen to combine a certain mechanical strength with biocompatibility, could be promising for a tendon *in vitro* model (Zeugolis et al., 2008a; Uquillas et al., 2012).

### 3D Bioprinting

In additive manufacturing, different processes are used to replicate 3D objects layer-by-layer by computer-aided design with controlled geometrical properties (Sculpteo, n.d.; De France et al., 2018; Vaezi et al., 2018). The conventional chemical engineering methods (as discussed above) fail to control exact pore size, pore geometry, and spatial distribution of the pores (Vaezi et al., 2018). Therefore, advanced additive manufacturing techniques are gaining popularity in recent tissue engineering strategies.

A popular technique is 3D bioprinting, in which a bioink is used to mimic the ECM and encapsulate the desired cells (Laternser et al., 2018). The most promising techniques are direct printing techniques, in which a 3D gel is directly printed in a single processing step (De France et al., 2018). The most common methods are micro-extrusion, inkjet and/or light−induced methods, which include laser−assisted bioprinting and stereolithography (Mandrycky et al., 2016). Bioprinting must be carefully prepared by collecting accurate tissue information, transferring information to a suitable computer-aided design model and, finally, by creating a stable structure (He et al., 2018). Specific demands for material and cell source have been listed: (i) the material should maintain cell viability and promote specific activity after printing, (ii) a great number of encapsulated cells needs to be available and (iii) cells should be able to survive the (post-)printing process (Zhang et al., 2017).

Extrusion-based 3D printing is a direct printing method which is widely researched and identified as an appropriate method for bone, cartilage and adipose tissue engineering. In contrast, this technique is still in its infancy for tendon tissue engineering, (Costantini et al., 2016; Sun et al., 2016; Naghieh et al., 2017; De Mori et al., 2018; Fernandez, 2019; Colle et al., 2020; Tytgat et al., 2020; Van Damme et al., 2020). For example, Sun et al. (2016) have demonstrated the overall superior qualities of 3D printed scaffolds over freeze-dried scaffolds for cartilage tissue engineering. Different extrusion-based bioprinters are designed to deposit various biopolymers, hydrogels and many different cell types to produce 3D bio-constructs (Vaezi et al., 2018). Merceron et al. (2015) developed a muscle–tendon unit construct, suitable as *in vitro* model by applying pneumatic pressure and heat to extrude filaments. The tendon part consisted of PCL co-printed with fibroblasts (NIH/3T3). The polymers were printed separately from the cell-laden bioinks by interspersing rows, and this process was repeated layer-by-layer with the whole construct being crosslinked at the end. After 7 days of culture, good cell viability was observed as well as the characteristic cell morphology. In addition, the construct was repeatedly produced with precise dimensional accuracy (Merceron et al., 2015). Laternser et al. (2018) described a novel drug screening platform for tendon and muscle applications. A tendon tissue model was designed by alternating layers of bioink and rat tenocytes in a dumbbell shape around postholder inserts in a microplate. The printed tenocytes showed high viability and maintained good differentiation, suggesting a good but rather basic approach for tendon drug screening (Laternser et al., 2018). Stanco et al. (2020) incorporated AT-MSCs in a nanofibrillar cellulose and alginate bioink for 3D printing, to evaluate tenogenic differentiation and suitability for tissue engineered constructs. The AT-MSCs survived the printing process and displayed the favorable tenogenic-like phenotype without an inflammatory response to the bioink. The research group reported a first approach for upscaling the clinical use of 3D printed tendon constructs, opening up possibilities for model design (Stanco et al., 2020). Park et al. (2018) designed a 3D bioprinted scaffold sleeve composed of PCL, PLGA and β tricalcium phosphate to reconstruct an anterior cruciate ligament. The scaffold was seeded with MSCs and evaluated in bone-tunnels of an *in vivo* rabbit model to assess bone-tendon regeneration. In the treatment groups, improved bone-tendon healing was observed at all time points and thus the authors concluded that their scaffold has the potential to accelerate bone-tendon healing in anterior cruciate ligament reconstruction (Park et al., 2018). Unfortunately, MSCs were still seeded afterward in this model instead of being encapsulated.

Recently, incorporating dECM in bioinks has attracted attention in the field of tissue engineering (Zhang et al., 2017; Santschi et al., 2019). Toprakhisar et al. (2018) developed a bioink from tendon dECM and evaluated murine fibroblast (NIH/3T3) viability and morphology. Vascularization, however, was not included in this model and cultures were only followed for 3 days. Furthermore, fibers were not properly aligned in contrast to fibers obtained with electrospinning. Regardless, the use of dECM in an *in vitro* model is limited due to the fact that its composition is variable and needs to be analyzed before every procedure.

Current drawbacks of direct bioprinting for tissue engineering applications include the low achievable sizes and the limited number of suitable materials (De France et al., 2018). When considering *in vitro* studies, the small scaffold size is not a constraint, but better fiber alignment has to be achieved and the impact of mechanical stimulation on 3D printed tendon constructs still needs to be evaluated.

The use of computer-aided design in bioprinting enables the combination with other fabrication techniques such as electrospinning. Jordahl et al. (2018) used 3D jet writing (a combination of electrospinning and 3D computer-aided design) to create bone constructs. Stability was obtained with PLGA and MSCs were seeded (Jordahl et al., 2018). In this approach, the benefits of both bioprinting and electrospinning were combined. When vascularization would be integrated in this model, this approach could be suitable for *in vitro* tendon models as well, providing a breakthrough in this research field.

## Advanced: Incorporating Bioreactors

**FIGURE 6:**
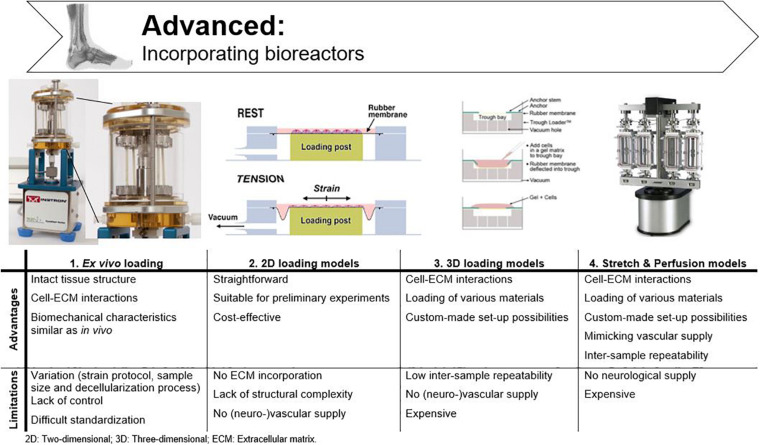
Advantages and disadvantages of bioreactors used in tendon tissue engineering. Adjusted from Ligagen^®^ L30–4C (DynaGen systems; Dartmouth, NS, Canada), BioFlex^®^ culture plates and TissueTrain^®^ (Flexcell International; Hillsborough, NC, United States), and BOSE BioDynamic^®^ 5,200 multi-chamber (TA Instruments; New Castle, United Kingdom).

Bioreactors are used to sustain the life of cells and tissues *in vitro*, while under the influence of dynamic, but controllable, physiological conditions (Youngstrom and Barrett, 2016; Ozbolat, 2017). Many different bioreactors have been used in tendon tissue engineering, as chronologically listed by Dyment et al. (2020). As already mentioned above, mechanical loading is essential to mimic tendon physiology, as cell signaling systems are modified through mechano-transduction pathways (Chiquet et al., 2009; Buxboim et al., 2010; Riehl et al., 2012; Govoni et al., 2017; Wang et al., 2017). Therefore, only bioreactors applying mechanical stimulation are discussed in this literature review, with the focus on parameters like mechanical stimulation, stability, and repeatability for fundamental research. A main drawback associated with bioreactors is that all researchers have used different protocols and it is challenging to compare studies. An overview of the cited bioreactors and corresponding stimulation protocols is given in Figure 6 and Table 1. Tension protocols ranged from 0 to 10% (magnitude) and a frequency between 0.0167 and 1 Hz.

**TABLE 1:** Overview of bioreactors used in tendon tissue engineering which all apply mechanical stimulation.

	Study	Biomaterial + Cells	Bioreactor	Stimulation	Results
***Ex vivo***	Angelidis et al., 2010	Decellularized rabbit hind paw Flexor tendon + AT-MSCs, fibroblasts	Ligagen L30–4C (DynaGen systems), clamped	Uniaxial strain, 1.25 N over 5 days. 1 cycle/minute in alternating 1h periods of mechanical loading and rest.	UTS and E comparable with fresh tendons. Cells reoriented parallel to the direction of the strain.
	Saber et al., 2010	Decellularized rabbit hind paw Flexor tendon + tenocytes	Ligagen L30–4C (DynaGen systems), clamped	Uniaxial strain, 1.25 N over 5 days. 1 cycle/minute in alternating 1 h periods of mechanical loading and rest.	UTS and E of loaded construct superior to non-loaded controls.
	Wang et al., 2013	Rabbit AT	Clamp grips, in medium	8 h/day, 0–9%, 0.25 Hz. 6 days	Loss of structure integrity and increased collagen III expression in unloaded tendons. 6% cyclic strain optimal for structure integrity and cellular function.
	Lee et al., 2013	Decellularized porcine anterior tibialis tendon	Vertically, in culture medium	10% tension, 1 Hz, 90° torsion. 7 days	20% lower UTS in decellularized grafts vs. normal tissue but doubled UTS after 7 days incubation
	Youngstrom et al., 2015	Decellularized equine SDFT + BM-MSCs	Horizontally clamp gripped, in medium	0%, 3%, 5% strain, 0.33 Hz, up to 1 h/day, 11 days.	Gene expression, elastic modulus and UTS favorable with 3%.
	Burk et al., 2016	Decellularized equine SDFT + AT-MSCs	Clamp grips, in medium	2% strain, 1 Hz, short (2 stretches/cycle) and long (3 stretches/cycle) protocol.	Short mechanical stimulation best cell alignment, successful tenogenic differentiation.
**2D loading**	Riboh et al., 2008	Rabbit tenocytes, sheath fibroblasts, BM-MSCs, AT-MSCs	UniFlex culture plate + Flexcell Tension System (Flexcell International)	Continuous strain (8%, 1 Hz). Intermittent strain (1 h on/5 h off, 4% 0.1 Hz).	Cell proliferation, collagen I production and tenocyte morphology increased with intermittent strain.
	Zhang and Wang, 2013	Mice tenocytes or TSPCs of AT or patellar tendon	Silicone dishes connected to stretching apparatus	12 h, 4% or 8%	Tenogenic gene expression increased in TSPCs with 4% mechanical stretching. Tenocyte and non-tenocyte related gene expression increased in TSPCs with 8% mechanical stretching. Tenocytes no strain-dependent response in non-tenocyte related gene expression.
	Gaspar et al., 2016	Human dermal fibroblasts, tenocytes, BM-MSCs + macromolecular crowding	MechanoCulture FX (CellScale Biomaterials Testing), clamp grips	12 h/day, 10%, 1 Hz	Cell/ECM alignment superior, increased ECM deposition and similar metabolic activity with mechanical loading.
	Gaspar et al., 2019	Human tenocytes, BM-MSCs, neonatal/adult dermal fibroblasts + macromolecular crowding	MechanoCulture FX (CellScale Biomaterials Testing), clamp grips	12 h/day, 10%, 1 Hz	Tenogenic phenotype maintained by tenocytes. No (*trans*)differentiation of BM-MSCs or fibroblasts.
**3D loading**	Altman et al., 2002	Collagen type I gel + bovine ligament fibroblasts, human BM-MSCs	Vertically oriented ligament growth between 2 anchors	Translational (10%, 2 mm) and rotational strain (25%, 90°). 0.0167 Hz (1 cycle of stress/relaxation per minute), 21 days.	Ligament markers upregulated, cell alignment/density increased and oriented collagen fibers.
	Garvin et al., 2003	Collagen type I gel + avian tenocytes	Tissue Train 3D Culture System (Flexcell International), culture plate with 2 anchors	1 h/day, 1% elongation, 1 Hz, 11 days	Tenogenic gene expression and linear morphology. Stronger loaded constructs vs. non-exercised controls.
	Scott et al., 2011	Collagen type I gel + mouse multi-potent mesenchymal cell line (C3H10T1/2)	Tissue Train 3D Culture System (Flexcell International), culture plate with 2 anchors	Static vs. cyclic load, 2 h/day, 5%, 0.1 Hz for 1, 2 or 3 weeks. 2 h/day, 0, 2.5, 5, 7.5, or 10%, 0.1 Hz for 2 weeks. 2 h/day, 10%, 0.1 Hz, 10,100, or 1,000 cycles/day, 10s rest	Tenogenic gene expression increased with cyclic loading. Gene expression increased with increasing magnitude, with 10s rest and increased repetitions.
	Jones et al., 2013	Collagen type I gel + human AT tenocytes	Tissue Train 3D Culture System (Flexcell International), culture plate with 2 anchors	5% cyclic uniaxial strain, 1 Hz, 48 h	Matrix metalloproteinases and tenogenic genes anabolically influenced.
	Bosworth et al., 2014	PCL + human BM-MSCs	BOSE BioDynamic chamber 5110 (TA Instruments), clamp grips	1 h/day, 5%, 1 Hz (3,600 cycles/day), 225 N, 7 and 21 days	Cell orientation more uniaxial, tendon gene upregulation due to dynamic loading.
	Wu et al., 2017	PCL/PLA scaffold + human tenocytes, AT-MSCs and HUVECs	MechanoCulture T6 Mechanical Stimulation System (CellScale Biomaterials Testing), clamp grips	2 h/day, 4%, 0.5 Hz, 12 days	Total collagen secretion upregulated, enhanced tenogenic differentiation with dynamic stretching.
	Atkinson et al., 2020	Collagen type I + equine tenocytes	Custom-designed bioreactor with clamps	20 min/day, 10%, 0.67 Hz, 14 days	Mechanical properties improved, more gel contraction by the tenocytes with loading.
**Stretch and perfusion**	Barber et al., 2013	Decellularized equine SDFT + rabbit BM-MSCs	Oscillating stretch-perfusion bioreactor, 6 separate chambers	3× 15–30–60 min of activity alternated with 15–30–60 min off, 2×/day. 3%, 0.33 Hz, 7 days. Perfusion: 100 μm/s	Collagen production and alignment superior in cyclic load vs. static culture.
	Hohlrieder et al., 2013	PLA nanofibers in yarns + human BM-MSCs	BOSE BioDynamic 5200 multi-chamber (TA Instruments), clamp grips	2 h/day, 10%, 1 Hz, 10 days Perfusion: 20 ml/min	Cytoskeleton realignment in fiber/applied strain direction, BM-MSCs adherence to fibers, tenogenic differentiation when differentiation medium + cyclic tensile strain.
	Xu et al., 2015	Braided silk fibroin + human ACL fibroblasts	Custom-made bioreactor, 10 independent reactor vessels, vertical movement	45° rotational and 3.5 mm translational deformations, 0.0667 Hz	Exact control of environmental conditions possible, load and stiffness of silk scaffolds matches native ACLs.
	Talò et al., 2020	PLA-PCL/Collagen scaffold + rat TSPCs	Custom-designed, in culture medium, loading plates	Cyclic tensile strain, 3 h/day, 2, 4, and 8 and 0.3, 0.5, and 1.0 Hz, 7 days	No difference in cell viability. Tenogenic gene expression highest with 4%, 0.5 Hz.

**ACL, anterior cruciate ligaments; AT, achilles tendon; AT-MSCs, adipose tissue-derived mesenchymal stem cells; BM-MSCs, bone marrow-derived mesenchymal stem cells; E, elastic modulus; HUVECs, human umbilical vein endothelial cells; PCL, poly-ε-caprolactone; PLA, polylactic acid; SDFT, superficial digital flexor tendon; TSPCs, tendon stem/progenitor cells; UTS, ultimate tensile strength*.*

To study tendon homeostasis under mechanical load, biomechanical parameters such as strength, toughness and viscoelasticity are evaluated (Murata, 2012). Other tensile properties for characterization of biomaterials are UTS, yield or failure load/stress/strain, and elasticity. While UTS displays the maximum stress before physical deformation disrupts the material irrevocably, the yield load/stress/strain displays the stress a material can tolerate before physical deformation occurs and the failure load/stress/strain illustrate the properties at the moment of failure (Malkin and Isayev, 2012b). Stress is the applied force over cross-sectional area. Strain, however, is a measure for the deformation in response to the applied stress. The ratio of stress over strain is called elasticity (or Young’s modulus) and illustrates how force results in deformation of the tissue (Malkin and Isayev, 2012a).

To allow cells to respond in an *in vitro* model similarly as *in vivo*, they should experience the physiological stiffness of the scaffold and undergo physiological levels of strain (Sensini and Cristofolini, 2018). The tendon response to mechanical load is varying depending on the location in the body, the age of the animal, and the differentiation level of the cells (Patterson-Kane et al., 2012). As different results are observed *in vivo* compared to *in vitro*, measurements cannot simply be extrapolated and should be evaluated ‘relatively’ (Smith and Goodship, 2008). For example, *in vivo* the strain of the SDFT in galop is measured 12-16%, while *in vitro* 15–17% is measured as ultimate tensile strain (Gerard et al., 2005; Thorpe et al., 2010). The effects of mechanical forces on MSC differentiation have been widely studied (Huang et al., 2009; Youngstrom et al., 2015; Youngstrom et al., 2016; Fahy et al., 2017). Depending on the mechanical stimulation protocol and MSC source used, differentiation into an osteogenic, chondrogenic, adipogenic, or tenogenic phenotype is observed (Delaine-Smith and Reilly, 2012). Therefore, the appropriate strain/stretch protocols for MSCs in a tendon environment should be identified in order to support tenogenic differentiation of the MSCs and, more importantly, the secretion of tenogenic trophic factors. In a study of Youngstrom et al. (2015) equine BM-MSCs were seeded on decellularized SDFT, subjected to 3 and 5% strain, 0.33 Hz for up to 1h daily and compared to static controls (0%). Upregulation of scleraxis expression was seen both in the 3 and 5% group compared to the control group, albeit only significant in the 3% group. Other evaluation criteria such as gene expression, elastic modulus, and UTS, were also superior in the 3% group. The 5% strain approach gave results in between the 0 and 3% strain group, leaning more toward the 0%, suggesting that 3% strain is more suitable for SDFT studies (Youngstrom et al., 2015). Human tenocytes cultured on rat tail collagen gels, on the other hand, expressed anabolic changes in matrix metalloproteinases and tenogenic genes when exposed to 5% strain, 1 Hz (Jones et al., 2013). In a study of Wang et al. (2013) in which rabbit Achilles tendons were subjected to 0–9% strain, 0.25 Hz for 8 h daily, 6% cyclic tensile strain was identified as optimal to maintain structural integrity and cellular functions (Wang et al., 2013). An electrospun PLA/PCL/collagen scaffold, seeded with rat tendon stem cells was subjected to cyclic tensile strain with different magnitudes and frequencies by Xu et al. (2015). The optimal protocol for enhancing tenogenic gene expression was determined as 4% and 0.5 Hz (Xu et al., 2015). Burk et al. (2016) seeded equine AT-MSCs on a decellularized tendon scaffold. When the scaffolds were exposed to 2% strain and 1 Hz, the viability decreased while tenogenic gene expression (tenascin-C and scleraxis) increased. Cell alignment was present on all scaffolds (static vs. cyclic stretching) and was more pronounced compared to monolayer cell cultures, indicating the importance of ECM mimicry (Burk et al., 2016).

Not only the amount of strain applied is significant, the regimen used is also of great importance. Continuous static loading not only affects tendon phenotype negatively, it is also not representative for the *in vivo* situation. Optimal for physiological mimicking is, therefore, the application of intermittent cyclic loading (Cao et al., 2006; Scott et al., 2011). Different studies show favorable cell characteristics (elongation, metabolic activity, gene levels and protein production) administering intermittent cyclic strain, while a negative influence of continuous cyclic strain is frequently reported (Cao et al., 2006; Riboh et al., 2008; Bosworth et al., 2014; Youngstrom et al., 2015; Atkinson et al., 2020). For example, Riboh et al. (2008) compared the application of constant versus intermittent cyclic strain in a 2D, Flexcell Strain Unit (Flexcell International; Hillsborough, NC, United States). Continuous cyclic strain inhibited cell proliferation and collagen production in tenocytes, sheath fibroblasts, BM-MSCs and AT-MSCs. Intermittent cyclic strain, on the other hand, increased cellular proliferation and total collagen production per cell (Riboh et al., 2008).

### *Ex vivo* Loading

To define mechanical characteristics of tendons, explants can be loaded onto bioreactors immediately after dissection or after decellularization. For defining biomechanical characteristics of *in vivo* tendons, *ex vivo* loading is the most appropriate method. However, mechanical properties vary due to the applied strain protocol, the size of the dissected tissue, and the decellularization process if applicable (Kuo et al., 2010; Youngstrom and Barrett, 2016). A custom bioreactor system (Ligagen L30-4C, DynaGen systems; Tissue Growth Technologies, Minnetonka, MN, United States) was used by two research groups to test the influence of oscillatory, uniaxial tensile stimulation on decellularized rabbit flexor tendons. Both reseeded the constructs with different cell types (AT-MSCs combined with fibroblasts, and tenocytes, respectively) and compared either seeded constructs to fresh tendons and unloaded constructs (Angelidis et al., 2010) or to unseeded tendons (Saber et al., 2010). Both studies found that the seeded tendon constructs showed mechanical characteristics (UTS and elastic modulus) comparable to those of freshly extracted tendon (Angelidis et al., 2010; Saber et al., 2010). Lee et al. (2013) evaluated porcine anterior tibial tendons for human ligament reconstruction in the bioreactor where the ligament was fixated vertically at both ends and tension up to 120% was applied while rotation was simulated also (0–90°). Decellularized grafts initially showed 20% lower UTS than normal tendon, but after 7 days of physical stimulation, the UTS was doubled, indicating that these constructs do have potential for human ligament reconstruction (Lee et al., 2013). Based on research with these *ex vivo* models, our knowledge about tendon physiological mechanics has substantially improved. Nevertheless, these models are too variable, and therefore, inadequate to study cellular responses *in vitro* (Wang et al., 2017).

### 2D Loading Models

As 2D models are still used to examine cell behavior [as discussed in section “Two-Dimensional (2D) Models”], implementing mechanical load is achievable in 2D loading bioreactors. Cells are cultured in modified cell-plates, allowing execution of a specified stretch protocol after cell adhesion to the bottom of the well. Both custom-made and commercial models are available and are usually placed within a standard incubator. The disadvantage of these 2D bioreactors is that they can only provide insights in cellular responses but not cell–matrix interactions (Wang et al., 2017; Yu et al., 2020). Zhang and Wang investigated tendon mechano-biological responses through different *in vivo* and *in vitro* experiments. For the *in vitro* experiment, mice AT/patellar tendon tenocytes and tendon stem cells were extracted, plated in silicone dishes and mounted on a custom-made stretching device (Wang et al., 2003). Their main finding was that a cell-dependent response occurred on the applied strain regimes. Tendon stem cells responded to moderate mechanical stretching (4%) with increased tenocyte-related gene expression (collagen I, tenomodulin). Exaggerated mechanical loading (8%) resulted in both increased tenocyte and non-tenocyte-related gene expression, suggesting differentiation into other cell types. Tenocytes, on the other hand, did not express this strain-dependent response. The researchers concluded that moderate mechanical loading could be beneficial in tendon homeostasis (Zhang and Wang, 2013). The combined effects of macromolecular crowding [where an attempt is made to create an optimal intracellular environment by the administration of various macromolecules, including different proteins and nucleic acids (Hata et al., 2018)] and mechanical loading in a commercially available MechanoCulture FX (CellScale, Biomaterials Testing; Waterloo, Canada) were evaluated by Gaspar et al. (2016) Increased alignment was seen together with increased ECM deposition, but with similar cell metabolic activity and viability. The same group later conducted a similar study using other cell types (tenocytes, BM-MSCs and dermal fibroblasts). While they successfully achieved maintenance of proper tenogenic phenotype by combining mechanical loading and macromolecular crowding, they failed to induce tenogenic differentiation of BM-MSCs and *trans*-differentiation of fibroblasts (Gaspar et al., 2019).

### 3D Loading Models

2D bioreactors are only suitable for loading monolayer cell cultures, therefore, a more sophisticated approach for 3D biomaterial scaffolds is needed (Yu et al., 2020). Various (bio-)materials can be loaded, and many alternative mount methods are available. For example, the end of the samples can be fixed with grips, but fixation between anchors is also possible. These reactors, however, lack vascularization, as the static presence of culture medium is not representative for *in vivo* blood flow. Additionally, culture medium is often individually provided per sample, limiting the inter-sample repeatability (Jaiswal et al., 2020). In the bioreactor of Altman et al., BM-MSCs seeded onto collagen I gel matrices were grown in 12 individual tubes in between two anchors (2 cm apart) for ligament tissue engineering. The complete system was designed to fit in a standard incubator. After application of translational and rotational strain, gene expression, cell alignment, and collagen fiber orientation suggested the BM-MSCs differentiated into ligament cells (Altman et al., 2002). Unlike some ligaments, tendons mostly experienced unidirectional stretching parallel to their orientation (Wang et al., 2017). The implementation of rotational strains is, therefore, unnecessary for tendon bioreactors. Garvin et al. (2003) inserted tendon constructs, made of a mixture of collagen I gel and tenocytes, in a commercially available Tissue Train 3D Culture System consisting of Tissue Train culture plates with two anchors (Flexcell International; Hillsborough, NC, United States). After 11 days of culture, constructs exposed to uniaxial displacement were stronger than unloaded counterparts, yet far weaker than native adult tendons (Garvin et al., 2003). The same bioreactor was used by Scott et al. (2011) to examine different strain protocols on bioartificial tendons, made of a cell-collagen mixture. Tenogenic gene expression (scleraxis and collagen I) in MSCs was increased by administering cyclic load (versus static load) with increasing magnitude (0, 2.5, 5, 7.5, or 10%), and with implementing 10s rest periods in between loading cycles and with increasing repetitions (10,100, or 1,000 cycles/day) (Scott et al., 2011). Bosworth et al. (2014) utilized the BOSE BioDynamic chamber 5110 (TA Instruments; New Castle, United Kingdom) for evaluating static (0%) versus cyclic loading (5% strain, 1 Hz for 1 h per day) on electrospun PCL yarns seeded with MSCs. Results showed increased cell proliferation, matrix deposition and gene expression in response to intermittent cyclic loading (Bosworth et al., 2014). Wu et al. (2017) cultured three different cell types (tenocytes, AT-MSCs and HUVECs) on synthetic electrospun fibers (PCL/PLA scaffolds) and stated that dynamic culture in a custom-made mechanical stimulation device promoted collagen production and tenogenic differentiation (Wu et al., 2017). Atkinson et al. (2020) demonstrated with the use of a custom-designed bioreactor that equine tendon constructs within collagen I gels, analogous to other species, show improved mechanics when exposed to cyclic strain (Atkinson et al., 2020).

### Stretch and Perfusion Models

As culture medium perfusion was incorporated in these bioreactors to mimic blood flow, these models are discussed separately. With the implementation of culture medium perfusion, cells have superior access to oxygen and nutrients which leads to increased proliferation rates (Wendt et al., 2006). Unidirectional perfusion is shown to create heterogeneous spreading of the cultured cells within 3D scaffolds, whereas bidirectional flow leads to more uniformly colonized scaffolds (Wendt et al., 2006; Du et al., 2009). Barber et al. (2013) braided nanofibrous scaffolds out of PLA and seeded them with BM-MSCs. Instead of replacing the differentiation medium every 2–3 days, active unidirectional perfusion (20 ml/min) was implemented (Barber et al., 2013). Hohlrieder and his group tackled the need to control exact environmental conditions to evaluate cell behavior. A new bioreactor was designed with 10 independent vessels to evaluate braided silk scaffolds for ligament graft design. Mechanical load and unidirectional perfusion were controlled per vessel (Hohlrieder et al., 2013). Talò et al. (2020) designed an oscillating stretch-perfusion bioreactor with programmable uniaxial strain for evaluating decellularized SDFT. Bidirectional perfusion was administered after MSC seeding, and the effect of stretching cycles was evaluated. The bioreactor has been declared unique and cost-effective for the incorporation of multiple chambers for controlling different biological and mechanical protocols (Talò et al., 2020).

Conclusive, different custom-made and commercially available bioreactors are being used with different mechanical loading protocols. The variety within these studies complicates translational research and hampers scientific progress (Beldjilali-Labro et al., 2018). For a universal tendon model, an optimal bioreactor with a multiple-chamber, multiple-sample set-up should be designed and used across laboratories, since different experimental conditions and appropriate controls are required (Jaiswal et al., 2020). The stretch-perfusion bioreactors are the most advanced and the most suitable for this application, but a universal loading protocol is lacking.

## Expert: Available Tendinopathy Models

**FIGURE 7:**
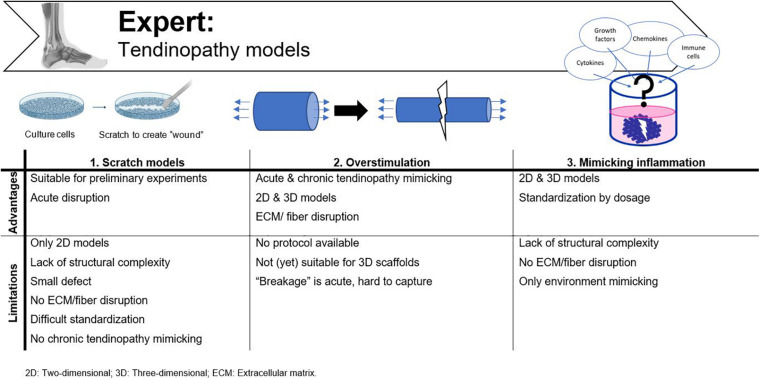
Advantages and disadvantages of tendinopathy models currently used. Adjusted from Reaction Biology Corp. (n.d.).

Two major techniques are available to provide a pathophysiological tendon model, namely scratch models and overstimulation-based approaches (Figure 7). To mimic the tendinopathy environment with its physical damage and mechanical disruption, researchers have supplemented various cytokines and enzymes to different *in vitro* cultures and animal models. It is evident, however, that when the physiological tendon model does not mimic *in vivo* tendon tissue regarding complexity and functionality, the “-pathy” models so far available are not very representative either. A main drawback is the lack of nutrition *in vitro*, i.e., vascular supply, to provide inflammatory cytokines and (immune) cells, which normally regulate the *in vivo* repair process (Dirks and Warden, 2011; Adekanmbi et al., 2017).

### Scratch Models

A popular way to assess cell motility in 2D cultures is by a scratch assay. A scratch defect can be created in confluent cell monolayers with a sterile pipette microtip and can then be monitored for several hours-days until closure of the scratch (Fessel et al., 2014). This technique is most frequently used in wound healing studies (Ranzato et al., 2009; Bussche et al., 2015; Liu et al., 2020), but can also represent an acute tendon injury (Fessel et al., 2014; Randelli et al., 2016; Adekanmbi et al., 2017). While *in vivo* all fibers are disrupted, in the scratch model only a small defect is made in the cell layers without the influence of ECM or fiber disturbance. Another issue with this method is standardization, as the defect is mostly applied manually and in contrast to the width of the scratch, which is more fixed as one size of pipette tips is chosen (e.g., a 2–200 μL pipet tip), trying to maintain a certain length is much more variable (Liang et al., 2007). Other culture-inserts [e.g., the Ibidi Culture-Inserts (Huang et al., 2019)] are available to perform more reproducible experiments. However, when these inserts are incorporated in a culture dish, cells grow separated by a cell-free gap and are not disrupted after applying a scratch, and as such, are less representative for tendon injury. Adekanmbi et al. (2017) created a tendon scratch model by simulating a tendon tear with a needle scratch on rat tail tendon fascicles to study the effect of high frequency, low magnitude loading. They standardized the scratch length with marker dots 1cm apart on the petri dish (Adekanmbi et al., 2017). Finally, the scratch model only represents an acute disruption, so no insights in chronic tendinopathy mechanisms can be evaluated, and these assays are usually performed in 2D models, lacking the complexity required for a fundamental *in vitro* model.

### Overstimulation

To mimic a tendinopathy and the accompanying inflammation response *in vitro*, overstimulation can be realized by applying a high mechanical load, a high stretch rate, or continuous cyclic duration. As there is currently no consensus on the optimal mechanical stimulation protocol for tendon homeostasis, it is difficult to define what is “too much.” Different studies report loading with excessive strain (>9%), which *in vitro* results in integrity damage, cell apoptosis and increased collagen III production (Legerlotz et al., 2013; Wang et al., 2013, 2017; Zhang and Wang, 2013). Dudhia et al. (2007) on the other hand, dissected the SDFT of horses of different ages to study the effect of aging on matrix metalloproteinases activity in a bioreactor with cyclic loading at 5% strain (1 Hz for 24 h). They concluded that their cyclic loading protocol decreased tendon tensile strength and function, and this effect was most pronounced in the group containing the older horses (19 years versus 3 years) (Dudhia et al., 2007). Wang et al. (2003) showed an increase in prostaglandin E_2_ secretion, an inflammatory mediator of tendinopathy, with increasing strain magnitude (4, 8, and 12%) in human tenocytes cultures on micro-grooved silicone dishes (Wang et al., 2003). Recently, Kubo et al. (2020) studied the short-term influence of 5% cyclic uniaxial stretching with a frequency of 1 or 2 Hz on primary tenocytes, cultured on PDMS chambers. The 1 Hz group expressed significantly more collagen I and had a higher cell proliferation compared to the 2 Hz or the non-stretched control group. Moreover, the group stimulated with 2 Hz displayed several metabolic changes, such as significant more cell apoptosis, reduced cell viability, and increased matrix metalloproteinase secretion. Their findings contribute to the identification of an appropriate overstimulation stretching profile (Kubo et al., 2020).

### Mimicking Inflammation

The actual impact of inflammatory processes on tendon disease is controversial. Whilst the early inflammation phase is clearly present in subacute injured tendons, persistent inflammation results in fibrosis and impaired healing. Various studies have evaluated supplementation with growth factors, cytokines, and chemokines to mimic the acute inflammatory phase of tendon injuries and to evaluate their effect on tenocytes, tendon stem/progenitor cells, and tenogenic differentiation of MSCs. An overview of all cells/inflammatory molecules involved in tendon pathophysiology is beyond the scope of this review and current insights have been recently reviewed elsewhere (Tang et al., 2018; Chisari et al., 2020). Briefly, interleukin -1β treatment reduced tenogenic gene expression of injured tendon-derived stem/progenitor cells (scleraxis, tenomodulin, collagen I, collagen III, biglycan and fibromodulin) and inhibited adipogenic, chondrogenic, and osteogenic differentiation of tendon stem/progenitor cells (Zhang et al., 2015). Stolk et al. (2017) studied the response of human tenocytes on pro-inflammatory factors and macrophages in an *in vitro* inflammation model, and reported altered surface marker and cytokine profiles. Furthermore, macrophage polarization was influenced by the inflammatory environment (Stolk et al., 2017). Alternatively, different immune cell populations can also be introduced in an *in vitro* at different time points, as these cells emerge depending on the stage of the disease. For example, and while there are no macrophages present in normal tendon tissue, pro-inflammatory M1 macrophages are observed in subacute injured tendons, whereas immunosuppressive M2 macrophages are mainly observed with chronic tendinopathy (Tang et al., 2018). Brandt et al. (2018) mimicked tendon inflammation by adding interleukin-1β or tumor necrosis factor-α, and evaluated the effect of peripheral blood leukocytes on AT-MSCs differentiation. High cytokine concentrations decreased ECM production and intracellular tenogenic gene expression in both monoculture and static culture conditions (co-cultures with leukocytes). More importantly, when dynamic loading was incorporated in the co-cultures, a reduced effect of the inflammation-mimicking cytokines was observed (Brandt et al., 2018). As tendinopathy pathophysiology is not yet completely understood and it is still unclear whether or not the onset of inflammation is triggered by immune cells or tenocytes, further research is mandatory to identify the inflammatory components which should be introduced into the disease model. In conclusion, a mixture of all the above-mentioned injury-mimicking mechanisms should be evaluated in 3D scaffolds.

## A State-Of-The-Art *in vitro* Tendinopathy Model

**FIGURE 8:**
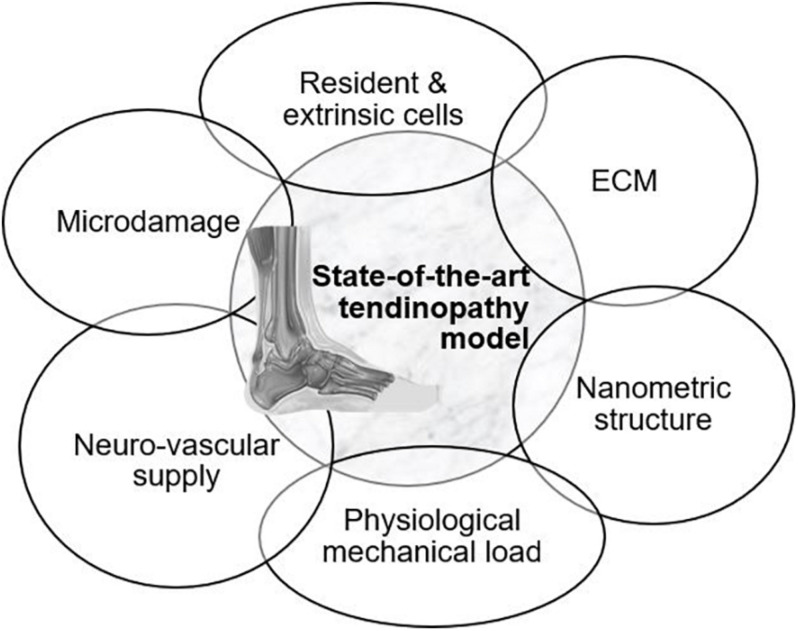
Requirements for the state-of-the-art tendinopathy model.

Based on the many requirements for a representative physiological tendon and/or pathological tendinopathy model *in vitro*, described throughout this review, it becomes clear that in order to establish a state-of-the-art tendinopathy model to improve knowledge on MSC-based tendon healing, an appropriate biomaterial should be colonized with a relevant cell source, combined with a pertinent production technology and subsequently cultured in the most suitable bioreactor (Caddeo et al., 2017). Mimicking the exact tendon pathophysiology *in vitro* is mandatory to (i) make significant progress in our understanding of tendinopathy mechanisms, (ii) unravel MSC-associated tendon healing, (iii) evaluate novel, regenerative treatments, and (iv) perform pharmacological experiments, while reducing the number of animals used for biomedical research purposes and related costs. The following requirements should be fulfilled: (i) representative cellular growth as demonstrated by the spindle-shape morphology and tenocyte marker expression, (ii) production of ECM and cell–matrix interactions, (iii) supporting nanometric and axially aligned structure (anisotropy), (iv) responsive to physiological levels of uniaxial strain, (v) neuro-vascular supply, and (vi) mimicking micro-damage like acute injuries and chronic overuse (Figure 8).

### Cells

The cells, relevant for the *in vitro* model, have to be capable of colonizing the scaffold similar to native tissue. In tendon tissue engineering, the most obvious cell type to utilize are tenocytes, as they are the most abundant cell type *in vivo* (Tan et al., 2015; Snedeker and Foolen, 2017; Wang et al., 2017; Schneider et al., 2018). Tenocytes can be easily isolated from adult tendons, but their unequivocal characterization is not evident as there is no unique tendon marker yet. Morphological characterization is usually performed, as tenocytes are spindle-shape, nicely aligned cells (Tan et al., 2015). It is generally accepted to confirm the tenogenic identity by demonstrating the presence of collagen I, collagen III, tenascin-C, scleraxis and tenomodulin, either on gene or protein level (Schweitzer et al., 2001; Zhu et al., 2010; Govoni et al., 2016; Wang et al., 2017). Furthermore, dedifferentiation of the tenocytes should be avoided by applying mechanical stimulation or other contact guidance cues (Nikolovski et al., 2003; Park et al., 2006; Kuo et al., 2010). Because adult cells have only a limited life span and have usually a low proliferation rate (Caddeo et al., 2017), another option is to use MSCs, which can be stimulated to differentiate into a tenocyte-phenotype (Lake et al., 2020). Similar to the *in vivo* situation, identifying the most appropriate cell source is challenging. BM-MSCs are believed to be the most suitable source to treat tendon injuries, but AT-MSCs or MSCs derived from neonatal sources are present in higher numbers and more easily accessible (Shojaee and Parham, 2019). Because tenogenic differentiation is not as straightforward to achieve and various stimulation parameters are involved, it is also possible to use the intrinsic stem cell population present in the tendon, called tendon stem/progenitor cells. These resident cells are responsible for tendon maintenance and repair. Furthermore, these cells are able to respond to inflammation following variable cytokine signals and growth factor profiles. When abnormal pathway activation occurs, tendon stem/progenitor cells differentiate into inappropriate cell types such as chondrocytes or adipocytes, resulting in calcification and metaplasia formation (Steinmann et al., 2020). Unfortunately, tendon stem cells are difficult to isolate because they are only present in small numbers *in vivo* (Wu et al., 2018).

In the healing process, both the intrinsic and extrinsic tendon cell populations, consisting of circulating cells and cells from nearby tissues, play a role. Therefore, to study the pathophysiological response in accordance to tendon injury, immunocompetent cells should also be incorporated in the model. Although the exact role of inflammatory and immune cells remains unclear (Jomaa et al., 2020), these cells are important for healing of the injured tendon (Caddeo et al., 2017; Tang et al., 2018; Nichols et al., 2019; Jomaa et al., 2020). Initial inflammation, accompanied by attraction of macrophages and neutrophils is crucial for healing. Pro-inflammatory M1 macrophages are mainly present during this early stage, but prolonged activity is reported to result in detrimental healing and increased scar tissue formation (Tang et al., 2018; Nichols et al., 2019). M2 macrophages, on the other hand, broadly described as anti-inflammatory, contribute to ECM deposition and remodeling. However, when inflammation and macrophage activity are completely inhibited, decreased mechanical properties of the healed tissue are observed. Nichols et al. (2019) reviewed the available research on cell populations during tendon healing phases, and frequently reported conflicting results. Therefore, inflammatory cell activity should first be clarified and subsequently balanced cell activity should be established *in vitro* to mimic the *in vivo* situation.

The interaction between resident cells (tenocytes, tendon stem cells, or MSCs), immune cells, growth factors and cytokines is crucial to gain new insights (Caddeo et al., 2017). These immunomodulatory cells/molecules are *in vivo* partially provided through the blood stream, therefore incorporation of vascular supply, or at least endothelial cells is mandatory (see section “Neuro-Vascular Supply”) (Tempfer and Traweger, 2015). Once the pathogenesis of tendinopathy is better understood, supplementing appropriate inflammatory signals and cells to the *in vitro* model to mimic the inflammation following injury, will further improve our insights in MSC healing mechanisms. In order to approximate the (patho-)physiological situation, one cell type will not be sufficient, but a balanced mixture of all of the above should be incorporated.

### Extracellular Matrix (ECM)

As stated above, tendon ECM consists of collagen I and III, which provides structure and mechanical strength. The proteoglycans (e.g., decorin and lumican) are important in fibrillogenesis and provide the tendon its high resistance to compressive and tensile forces (viscoelasticity) (Tan et al., 2015; Schneider et al., 2018). For an *in vitro* model, ECM should be included as it represents 80% of the *in vivo* tendon composition. Furthermore, cell–matrix interactions should be enabled as ECM contains many different growth factors, cytokines, and chemokines (Kleinman et al., 2003; Buxboim et al., 2010). Simulating ECM using natural materials is the most straightforward way to establish a representative *in vitro* tendon model, but highly modified synthetic materials (e.g., after adding integrin-binding peptide sequences) represent a valuable alternative.

### Supporting Nanometric Structure

There are three important matrix scales in tissue engineering: the macroscopic shape (cm–mm level), the pore structure regulating cell invasion and cell growth (μm level) and surface chemistry which controls cell adhesion and gene expression (nm level) (Kim and Mooney, 1998). For tendon tissue, this implies that fiber diameters should be varying between 2 μm, i.e., diameter of collagen fiber is 1–20 μm, and 200 μm, i.e., diameter of fascicles (Figure 1) (Liu Y. et al., 2008). When murine fibroblasts (C3H10T1/2) were cultured on fibers with larger diameter (>2 μm), an increased tenogenic gene expression was observed when compared to small (<1 μm) and medium fiber diameters (1–2 μm) (Cardwell et al., 2014). The maximum fiber diameter is mainly limited by oxygen diffusion capacity (max. 200 μm). *In vivo*, fascicles are surrounded by endotenon (Figure 1), which includes blood vessels providing nutrients and oxygen, and thus, the designed *in vitro* scaffolds also need to support nutrients and oxygen supply (Kim and Mooney, 1998; Lu et al., 2013). Furthermore, pore size is also critical, as it allows cell infiltration, nutrition, proliferation and migration (Sensini and Cristofolini, 2018; Luo, 2020). Besides superior cell characteristics, increased and interconnected porosity (usually >90%) also facilitates efficient nutrient and oxygen diffusion and waste removal (Loh and Choong, 2013). The lower limit of pore size is determined by cell size (±20 μm) whereas the upper limit is depending on biomaterial and cell combination (100–200 μm) (Freyman et al., 2001). However, the disadvantage of high porosity is the fact that mechanical properties are compromised due to the large amount of dead volume (Loh and Choong, 2013).

Because of the axially aligned nature of collagen fibrils *in vivo*, a tendon model should provide parallelly aligned fibers (Cardwell et al., 2014) to provide tensile strength during loading and cell proliferation stimuli (Kleinman et al., 2003; Dudhia et al., 2007). The microstructural anisotropy might even influence gene expression levels without modifying gene sequence (epigenetic role) (Wang et al., 2018). Therefore, an *in vitro* tendinopathy model should be designed by implementing electrospinning and extrusion-based 3D printing.

### Physiologically Relevant Mechanical Load

**FIGURE 9:**
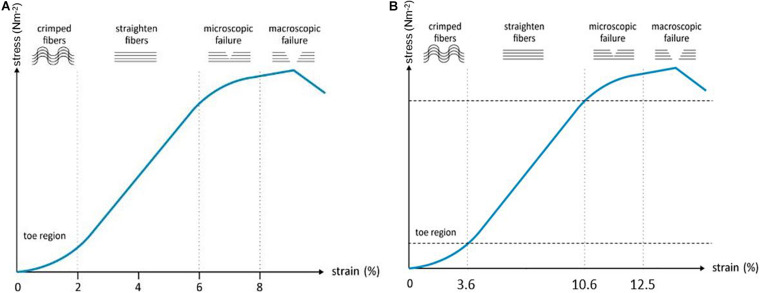
**(A)** Stress-strain curve of the human Achilles tendon. **(B)** Stress-strain curve of the equine superficial digital flexor tendon. Stress: applied force over area, Strain: deformation in response to stretch, Elasticity: stress/strain ratio or how force results in a deformation of the tissue. Adjusted from Barfod (2014).

The hierarchical anisotropic tendon structure typically enables its non-linear mechanical properties (Sensini and Cristofolini, 2018). When tendons are subjected to mechanical load, the collagen fibers stretch out, nullifying the unique crimp-pattern (called “toe region”). When the mechanical load is increased further, collagen molecules become more aligned, resulting initially in linear stretching, followed by microscopic damage and finally macroscopic tearing (Wang, 2006; Sensini and Cristofolini, 2018). The slope of the stress-strain curve represents the tendon stiffness, also known as Young’s modulus. The linear phase of this curve in human tendons is quite short (2–6%) and similar for each tendon of the human body (Figure 9). The loading response of the equine SDFT results in a similar stress-strain curve with linear deformation between 3.6 and 10% (examined on an adult fore limb SDFT *in vitro*) which supports the claim for the equine SDFT as a model for human Achilles tendinopathy (Figure 9) (Patterson-Kane et al., 2012; Sensini and Cristofolini, 2018). Mechanical properties, however, are dependent on tendon cross-section and function (Sensini and Cristofolini, 2018). As the aim of the *in vitro* model is to generate a physiological response, the cultured cells should experience scaffold stiffness and strain comparable to the *in vivo* situation, resulting in similar cell behavior (Patterson-Kane et al., 2012).

As musculoskeletal loading is essential for maintaining tendon homeostasis, cyclic strain should be included in culture systems (Patterson-Kane et al., 2012). While subtle changes in mechanical loading result in an anabolic and anti-inflammatory response, both over- and under-stimulation can result in degeneration and remodeling of the ECM (Patterson-Kane et al., 2012; Wang et al., 2013; Youngstrom and Barrett, 2016). As already mentioned, it is challenging to identify a suitable protocol to mimic the *in vivo* load of tendon. Indeed, the tendon response to mechanical load is varying depending on the location in the body, the age of the human or animal, and the differentiation level of the cells (Patterson-Kane et al., 2012). In conclusion, mechanical loading should be within physiological range, i.e., (Achilles tendon: 2–6%, SDFT: 3–10%, Figure 9) in the context of translational research.

### Neuro-Vascular Supply

*In vivo*, tendon is considered as a hypovascular tissue as blood supply is only present in the endo- and epitenon (Evans, 2012; Docheva et al., 2015; Tempfer and Traweger, 2015; Schneider et al., 2018; Sensini and Cristofolini, 2018; Costa-Almeida et al., 2019). Nevertheless, also in tendon, cells, growth factors and cytokines should be delivered through the blood stream, besides nutrients and oxygen. Increased vascularization and scar tissue formation is observed with tendon injuries (Tempfer and Traweger, 2015). Therefore, vascularization should also be incorporated in an *in vitro* tendinopathy model which remains a major challenge in tissue engineering. 3D bioprinting, however, could offer a solution. As proposed by Richards et al. (2016), a mixture of tissue-specific and pro-vascular bioink should be printed with tenocytes and endothelial cells in exact spatial distribution. Wu et al. (2017) showed that the expression of tenogenic markers was upregulated when AT-MSCs, tenocytes, and HUVECS, were cultured together, illustrating that tenocytes also profit from providing vascularization.

Besides blood vessels, the endo- and epitenon contain nerves and lymphatic vessels to support the tendon cells in their function (Wang, 2006; Docheva et al., 2015; Tan et al., 2015; Schneider et al., 2018). During tendon inflammation, different neuronal mediators play an active role in regulating pain and the inflammation process (Ackermann, 2013; Tempfer and Traweger, 2015). However, the exact mechanisms are not yet elucidated. The incorporation of (induced) pluripotent stem cells capable of neural differentiation and MSCs or adult neural stem cells, in an *in vitro* model is of great importance (Maltman et al., 2011; Azari and Reynolds, 2016; Snedeker and Foolen, 2017), but is still in its infancy for both tendon and other types of tissue engineering.

### Microdamage Leading to Failure

It is generally accepted that tendinopathy occurs due to chronic overuse. As a reaction to the matrix disruption, *in vivo* neovascularisation is observed. VEGF is highly secreted which results in increased matrix metalloproteinase secretion and further matrix degradation (Tempfer and Traweger, 2015). Petersen et al. (2004) demonstrated increased VEGF secretion in response to 1 Hz cyclic stretching and decreased expression if low frequency is used (0.5 Hz). As discussed above, by subjecting strain above physiological level, one should be able to create a tendinopathy model (Wang et al., 2003, 2013). The exact protocol should be defined as different responses will be observed depending on the (bio-)material of choice. Incorporation of inflammatory molecules and cells will represent the detrimental environment after the ideal mechanical protocol is defined.

## Conclusion

After decades of research, it is clear that MSC-derived bioactive factors have great regenerative potential in healing tendon injuries. Yet, clinical application remains limited due to the unclarified pathogenesis of tendinopathy and MSCs’ underlying mechanisms of action. The complexity of *in vitro* tendon engineering has been evolving the last decade, and substantial progress has been made in mimicking tendon physiology by switching from 2D tenocyte cultures to 3D jet writing. The appropriate biomaterials, bioreactor and production technology should be combined to create *in vitro* tendon (Govoni et al., 2016). However, currently used models are far from ideal, especially by the functional (neuro-)vascular supply which is still lacking (Richards et al., 2016). The major remark in all listed models is the lack of consistency. All research groups developed and used their own protocols, mono-materials and hybrids, making conclusions impossible and preventing scientific progress. Moreover, both natural and synthetic materials are used interchangeably, in different combinations, and in different proportions. A crucial step to success is identifying the optimal mix of materials, manufacturing techniques, and biological stimuli, to mimic representative tendon properties. To achieve this, a multidisciplinary approach is needed where experts in biotechnology, cell biology, molecular biology, material sciences, physics, physiology, bioengineering and polymer chemistry join forces (Siemionow, 2015; Aibibu et al., 2016). We propose future models need to incorporate collagen or gelatin simulating the tendon ECM, either chemically modified or reinforced with a strong synthetic material, such as PCL, to mimic the cellular microenvironment (ECM, mechanical characteristics, …). As cell encapsulation is the most representative technique, resident cells (tenocytes, tendon stem cells, or MSCs), immune cells, growth factors and cytokines should be incorporated in a biomaterial-cell mixture for 3D-bioprinting. Applying mechanical stimulation in a stretch-perfusion bioreactor, once the appropriate strains have been identified, will enable to representatively mimic the tendon environment in both physiological and pathological conditions. In addition, vascularization should be present either by medium perfusion or by incorporating endothelial cells. A representative pathophysiological tendon model can be established by combining mild overstimulation for a longer period of time (e.g., 3 weeks), mimicking the chronic situation, and an acute extreme overloading and/or scratch, representing the acute injury. It is only a matter of time until tendon pathophysiology is unraveled as tendon tissue engineering strategies are rapidly evolving. Therefore, *in vitro* models can provide strategies to solve the MSC puzzle and evidence-based treatment protocols can be established which will quickly become available to tendinopathy patients, irrespective of the species.

## Author Contributions

MM performed the bibliographic research, drafted the manuscript, and created the figures. GV, SV, and CD revised the manuscript and supervised the process. CD finalized the manuscript. All authors contributed to the article and approved the submitted version.

## Conflict of Interest

The authors declare that the research was conducted in the absence of any commercial or financial relationships that could be construed as a potential conflict of interest.

## Abbreviations

2D: two-dimensional
3D: three-dimensional
AT-MSC(s): adipose tissue-derived mesenchymal stem cell(s)
BM-MSC(s): bone marrow-derived mesenchymal stem cell(s)
dECM: decellularized extracellular matrix
ECM: extracellular matrix
ELAC: electrochemically aligned collagen
HUVEC(s): human umbilical vein endothelial cell(s)
MSC(s): mesenchymal stem cell(s)
PCL: poly- ε-caprolactone
PDMS: polydimethylsiloxane
PGA: polyglycolic acid
PLA: polylactic acid
PLGA: polylactide-co-glycolide
SDFT: superficial digital flexor tendon
UTS: ultimate tensile strength
VEGF: vascular endothelial growth factor.
